# Predicting Stress–Strain Curve with Confidence: Balance Between Data Minimization and Uncertainty Quantification by a Dual Bayesian Model

**DOI:** 10.3390/polym17040550

**Published:** 2025-02-19

**Authors:** Tianyi Li, Zhengyuan Chen, Zhen Zhang, Zhenhua Wei, Gan-Ji Zhong, Zhong-Ming Li, Han Liu

**Affiliations:** 1 SOlids inFormaTics AI-Laboratory (SOFT-AI-Lab), Sichuan University, Chengdu 610065, China; 2 College of Polymer Science and Engineering, Sichuan University, Chengdu 610065, China; 3 College of Mathematics and Physics, Chengdu University of Technology, Chengdu 610059, China; 4 Department of Ocean Science and Engineering, Southern University of Science and Technology, Shenzhen 518055, China; 5 State Key Laboratory for Polymer Materials Engineering, Sichuan University, Chengdu 610065, China

**Keywords:** polymeric materials, mechanical behavior, machine learning, prediction uncertainty, polymer processing

## Abstract

Driven by polymer processing–property data, machine learning (ML) presents an efficient paradigm in predicting the stress–strain curve. However, it is generally challenged by (i) the deficiency of training data, (ii) the one-to-many issue of processing–property relationship (i.e., aleatoric uncertainty), and (iii) the unawareness of model uncertainty (i.e., epistemic uncertainty). Here, leveraging a Bayesian neural network (BNN) and a recently proposed dual-architected model for curve prediction, we introduce a dual Bayesian model that enables accurate prediction of the stress–strain curve while distinguishing between aleatoric and epistemic uncertainty at each processing condition. The model is trained using a Taguchi array dataset that minimizes the data size while maximizing the representativeness of 27 samples in a 4D processing parameter space, significantly reducing data requirements. By incorporating hidden layers and output-distribution layers, the model quantifies both aleatoric and epistemic uncertainty, aligning with experimental data fluctuations, and provides a 95% confidence interval for stress–strain predictions at each processing condition. Overall, this study establishes an uncertainty-aware framework for curve property prediction with reliable, modest uncertainty at a small data size, thus balancing data minimization and uncertainty quantification.

## 1. Introduction

**Figure 1:**
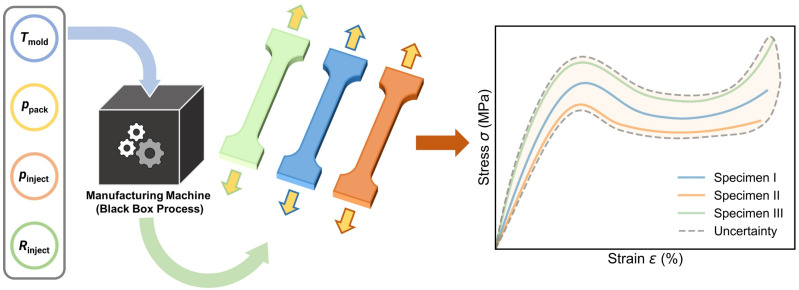
Schematic of the stress–strain curves illustrating the aleatoric uncertainty associated with polymer specimens prepared under identical injection molding conditions. The three-colored curves represent three separate specimens, highlighting the inherent variability in mechanical behavior, even when processed with the same parameters (i.e., mold temperature *T*_mold_, packing pressure *P*_pack_, injection pressure *P*_inject_, and injection rate *R*_inject_) due to the complex, black box nature of the polymer manufacturing process.

Due to their structural complexity, polymeric materials typically exhibit intricate stress–strain curves. Additionally, the “black box” simulation process (as shown in Figure 1) during the manufacturing stage poses a challenge for accurate prediction through physics-driven simulations [1]. As an alternative, machine learning (ML) provides an efficient approach to “bypass physics laws” and extract the polymer processing–property relationship purely from training data [2], enabling accurate prediction of stress–strain curves at different processing conditions [1,3,4,5,6,7]. However, ascribed to (i) its data-driven nature and (ii) the curve-output complexity, the ML approach is generally limited by the deficiency of training data size [8], failing to extract a reliable correlation pattern between polymer processing conditions and their resultant specimens’ stress–strain curves, with no confidence interval attached to each prediction [9,10,11]. Moreover, considering the intrinsic and inevitable fluctuations at each processing condition, the specimens prepared at the same condition generally show some variations in stress–strain curves, that is, the “one-to-many” issue [1,12], or referred to as aleatoric uncertainty (see Figure 1), which apparently falls out of the ML applicability of the “one-to-one” training scheme that requires unique mapping between inputs and outputs [13]. As such, it presents a grand challenge for ML approaches to address the “one-to-many” issue and predict stress–strain curves with confidence at a small data size.

By incorporating physics principles as prior knowledge, recent ML models have been demonstrated to hold the promise to greatly reduce the needs for large training data size [14,15,16]. Impressively, by integrating a curve type classifier and a curve feature regressor, a dual neural network (DNN) model is recently proposed to enable stress–strain curve prediction at an extremely small data size [1]. In contrast, using a single model to predict the entire stress–strain curve vector would significantly increase model complexity and require much larger amounts of training data. Despite their reduced data-size requirement, the ML models are mathematically incapable of addressing the “one-to-many” issue and outputting different stress–strain curves simultaneously at one processing condition—that is, “aleatoric uncertainty” [17], and little is known about the confidence interval of model prediction without quantifying the uncertainty of model parameters—that is, “epistemic uncertainty” [9,17]. In that regard, various statistical methods have been developed to establish uncertainty-aware ML models, with both aleatoric and epistemic uncertainty included [18]. Specifically, Bayesian neural networks (BNNs) have been a representative methodology for uncertainty quantification and, by providing sufficient training data [19,20], have been applied to successfully predict simple-patterned stress–strain curves with a 95% confidence interval provided for reliability guidance [21,22]. However, heavily relying on a large data size, the BNN models would generate unrealistically wide uncertainty estimations at a small data size, thus failing to evaluate the prediction reliability. Obviously, it is challenging to balance data minimization and uncertainty quantification, and as a result, there remains a lack of ML models to predict the stress–strain curve with reliable modest uncertainty at a small data size.

Here, combining (i) the BNN model for uncertainty quantification and (ii) the DNN model for curve prediction at a small data size [1], we introduce a dual Bayesian model to predict the stress–strain curve with reliable modest uncertainty at a small data size by taking the example of injection-molded polypropylene specimens prepared at different molding conditions. Inherited from the DNN model, the present architecture features the state-of-the-art simplicity of 183 neurons in total in the hidden layers for stress–strain curve prediction, significantly reducing the need for extensive training data. The construction of training data adopts a Taguchi array dataset of 27 samplings in a 4D processing parameter space, that is, a small dataset evenly distributed across the space to capture all main features of property evolution as a function of processing parameters (see Section 3.1). Based on the DNN architecture setting and Taguchi sampling strategy, the model further adds a probability density distribution layer as the output in order to mathematically address the “one-to-many” issue and quantify the aleatoric uncertainty accordingly (see Section 3.2). By assuming normal-distribution-type uncertainty, the present model yields normal distributions in good agreement with the training data distributions of curve features at different molding conditions, with an average miscalibration area ≈ 0.11 under its uncertainty calibration curve (see Section 3.3). Finally, in order to evaluate its prediction reliability, the model’s epistemic uncertainty is quantified by adopting BNN hidden neurons to establish a *prior* normal distribution for each model parameter, that is, neuron weights and biases. After training these parameter distributions, the model estimates the epistemic uncertainty of its output distribution by statistically analyzing the outputs at different model parameters (see Section 3.4). Notably, the incorporation of epistemic uncertainty leads to an increased but yet satisfactory modest 95% confidence interval of stress–strain curve prediction, with an average miscalibration area ≈ 0.18 for each curve feature, demonstrating the model’s applicability for reliability guidance (see Section 3.5). Overall, this study pioneers an uncertainty-aware dual Bayesian framework for stress–strain curve prediction (or curve property in general) with reliable modest uncertainty at a small data size, thus balancing data minimization and uncertainty quantification.

## 2. Materials and Experimental Methods

### 2.1. Materials

The isotactic-polypropylene (iPP) granules used in this study are of the commercial type “T30S” iPP, manufactured with Ziegler-Natta catalysts and procured from Yanchang Petroleum Refining and Petrochemical Company in Xi’an, China. These granules have a melting temperature of 161.2 °C, as determined by differential scanning calorimetry (Q2000, TA Instruments-Waters LLC, New Castle, DE, USA), and a melt flow rate of 3.2 g/10 min (2.16 kg and 230 °C).

### 2.2. Injection Molding Experiments

**Figure 2:**
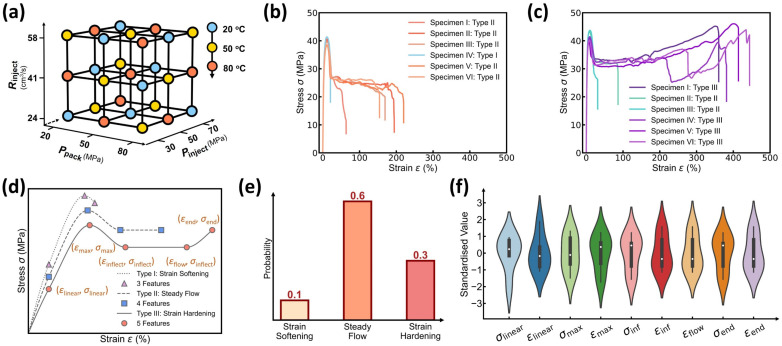
Dataset visualization of the stress–strain curves at different molding conditions. (**a**) Selected 27 molding conditions with the Taguchi method. Each condition has four tunable parameters, including mold temperature Tmold, packing pressure Ppack, injection pressure Pinject, and injection rate Rinject. (**b**) Stress–strain curves of specimens prepared at Tmold = 80 °C, Ppack = 20 MPa, Pinject = 70 MPa, and Rinject = 24.858 cm^3^/s. (**c**) Stress–strain curves of specimens prepared at Tmold = 20 °C, Ppack = 20 MPa, Pinject = 30 MPa, Rinject = 24.858 cm^3^/s; (**d**) Schematic illustrating three different stress–strain curve types, including strain softening type, steady flow type, and strain hardening type. The points indicate key curve features, including linear limit point, maximum yielding point, strain softening inflection point, steady flow limit point, and fracture point. (**e**) Example of curve type distribution at one molding condition. (**f**) Violin plot of the distribution of standardized curve features at one molding condition.

Employing a commercial hydraulic injection machine obtained from Haitian Plastics Machinery Ltd. Company in Ningbo, China, the iPP granules are injection-molded into dumbbell-shaped specimens with dimensions of 30 mm (length), 5 mm (width), and 1 mm (thickness) during the injection molding process, where four key tunable parameters determine the final mechanical performance of material. These parameters encompass (i) injection pressure ($P_{inject}$), (ii) injection rate ($R_{inject}$) for mold filling, (iii) packing pressure ($P_{pack}$) to sustain cavity filling post 95% mold occupation, and (iv) mold temperature ($T_{mold}$) during the filling and cooling stages as indicated in Figure 1; the detailed data have been screened using the Taguchi method and are thoroughly illustrated in Section 3.1, along with Figure 2a. While maintaining other pertinent factors constant, such as a 5 s packing time, a 30 s cooling time, and a processing temperature gradient along the injection screw (sequentially set as 160 °C, 180 °C, 200 °C, 210 °C, and 200 °C from hopper to nozzle), the specimens are prepared under diverse molding conditions within the operable range.

### 2.3. Stress–Strain Curve Measurements

Tensile tests are performed to acquire stress–strain profiles until fracture for all injection-molded iPP samples (oriented longitudinally, parallel to the injection flow). These tests are executed using the “Instron Model 5576 Series” Universal Testing System (Norwood, MA, USA) in adherence to the American Society of Testing and Materials (ASTM) D-638 testing standards, with testing conditions set at a room temperature of 23 °C and a tensile speed of 50 mm/min.

## 3. Result

### 3.1. Description of the Stress–Strain Curve Dataset

(1) Mini-Dataset Construction by Taguchi Sampling Representation

To build a small-sized but high-quality dataset, we utilize herein the Taguchi orthogonal method, a robust experimental design technique that minimizes the number of experiments needed to create a mini-dataset of 27 molding conditions (see Figure 2a) [1,23]. Each condition with four adjustable processing parameters, namely, $\left\{P_{inject},\;R_{inject},\;P_{pack},\;T_{mold}\right\}$, varies two parameters from surrounding conditions using three levels of alteration in the 4D processing parameter space, with orthogonal arrays systematically ensuring even distribution of experiments across the entire parameter space and providing an informative representation of the stress–strain curve evolution as a function of processing parameters. For each of the 27 molding conditions, 3 to 7 iPP injection-molded specimens are used to characterize the aleatoric uncertainty of stress–strain curves at each condition or to ensure their statistical replicability, resulting in a total of 152 curves in the dataset. This Taguchi sampling strategy is capable of covering the entire 4D design space with an extremely small yet highly informative dataset. As a *posteriori* validation, the resultant model predictivity would manifest the Taguchi-guided dataset’s high quality (see Section 3.3).

(2) “One-to-Many” Curve Variation at Each Molding Condition

We now take a close inspection into the stress–strain curve patterns at each molding condition. Figure 2b,c show two example sets of iPP specimens’ stress–strain curves at two molding conditions, respectively, wherein the curve patterns follow the typical mechanical behaviors of semicrystalline polymers and exhibit complex multiple regimes [24,25,26,27,28,29], including strain softening regime, steady flow regime, and strain hardening regime (see Figure 2d), governed by iPP’s complex microstructural evolution. Notably, at each molding condition, the stress–strain curves exhibit high aleatoric uncertainty and show an extent of fluctuation—especially for the elongation at break—that is, the “one-to-many” issue, which is ascribed to the intrinsic and inevitable fluctuations of processing parameters and the specimens’ microstructural discrepancy thereof. To address the “one-to-many” issue, the curve variation at each molding condition would be simplified into a dual distribution representation for DNN model construction (see Section 3.2 and Section 3.3).

### 3.2. Simplifying the “One-to-Many” Variational Curve Representation by a Dual Distribution

(1) Categorical-Distributed Nature of Curve Type

Since ML model training strictly follows the principle of “one-to-one” unique mapping between inputs and outputs [13], we address herein the issue of “one-to-many” curve variation at each molding condition by simplifying each set of stress–strain curves at one condition as a dual distribution—that is, a coupled distribution of curve types and features, ready to reconstruct the expected stress–strain curve and its aleatoric uncertainty (see Section 3.3). Accordingly, the ML model would output a dual distribution that describes the curve variation at each molding condition, rather than simultaneously outputting the many different curves themselves that would be out of the model’s capability.

Regarding the dual distribution, we first investigate the categorical distribution of curve type at each molding condition. Based on the variation in iPP’s mechanical response, the curve type can be categorized into three groups, as illustrated in Figure 2d, as follows: (i) Type I, which is characterized by strain softening; (ii) Type II, which exhibits steady flow; and (iii) Type III, which displays strain hardening after steady flow. Notably, the stress–strain curves at one molding condition generally have the same curve type or exhibit a dominant curve type with a few exceptions, as shown in Figure 2b,c. Further, Figure 2e shows an example of curve type distribution at one molding condition, which covers all three curve types with one curve type dominating, highlighting the categorical distributed nature of curve type at each molding condition. Accordingly, this probabilistic distribution would be forecasted by constructing an ML classifier configured with a categorical distribution output layer (see Section 3.3).

(2) Approxi-Normal-Distributed Nature of Curve Feature Point

Next, we investigate the distribution of curve features associated with five feature points that dictate the evolution trend of stress–strain curves, as illustrated in Figure 2d, including (i) the linear limit point ($\varepsilon_{linear},\;\sigma_{linear}$), which marks the end of the linear change of the curve; (ii) the maximum yielding point ($\varepsilon_{max},{\;\sigma }_{max}$), where the curve meets the first peak and the slope turns from positive to negative; (iii) the strain softening inflection point ($\varepsilon_{inflect},\;\sigma_{inflect}$), where the curve curvature starts to change from negative to positive; (iv) the steady flow limit point ($\varepsilon_{flow},\;\sigma_{inflect}$), which has the same stress value but more elongation; and finally, (v) the fracture point ($\varepsilon_{end},{\;\sigma }_{end}$) when the curve ends. Note that, instead of predicting the entire curve points, predicting solely the five feature points—together with the dominant curve type information—is ready to reconstruct the corresponding stress–strain curve (see Section 3.3), thus significantly reducing the ML model complexity and the data size requirement.

Figure 2f shows a violin plot of the distribution of curve features at one molding condition, wherein the white point indicates the median, the shape’s width reflects data frequency, and each feature has been standardized for illustration purposes. To a moderate extent, all curve features share more or less similar distribution characteristics, with an approxi-symmetric or slightly skewed peak centered around zero, highlighting the approxi-normal distributed nature of curve features. For simplicity, these curve feature distributions would be forecasted by constructing an ML regressor configured with a normal distribution output layer (see Section 3.3), that is, approximating each curve feature’s aleatoric uncertainty by a normal distribution.

### 3.3. Predicting the “One-to-Many” Variational Curve by a Dual-Distribution Neural Network

(1) Dual-Distribution Neural Network Architecture

**Figure 3:**
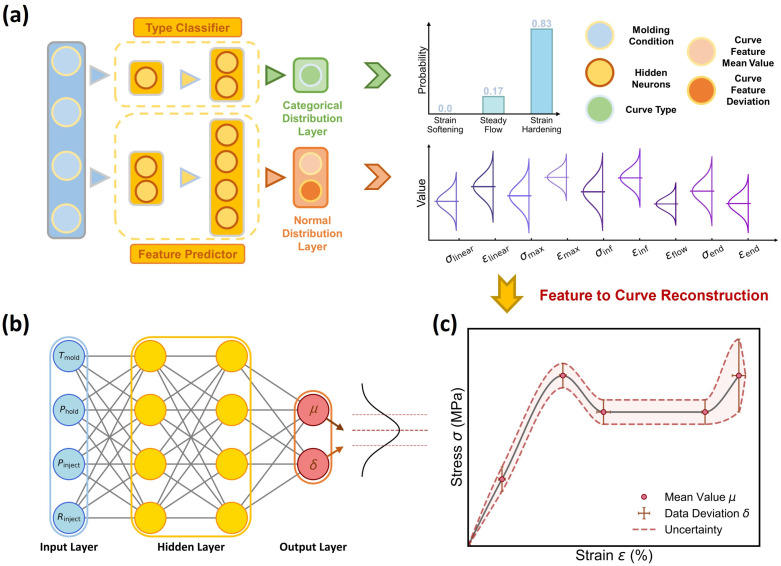
Schematic of the dual-distribution neural network (DNN). (**a**) DNN architecture built to predict the dual-distribution representation of curve variation, including (i) the categorical distribution of curve type and (ii) the approxi-normal distribution of each curve feature, provided by a curve type classifier and a curve feature predictor, respectively. (**b**) Schematic of the curve feature predictor, which outputs each feature’s aleatoric uncertainty as a normal distribution represented by its mean *μ* and standard deviation *δ*. (**c**) Schematic of the reconstructed stress–strain curve and its aleatoric uncertainty based on the predicted curve type and feature distribution.

To predict the dual-distribution representation of curve variation at each molding condition, we construct herein a dual-distribution neural network (DNN) model. Figure 3a shows the DNN model architecture built by a parallel integration of a curve-type classifier and a curve feature regressor, which takes molding condition as input and is responsible for outputting a distribution of curve type and feature, respectively. Note that, regarding the output-distribution format, the curve-type classifier adopts a categorical distribution output layer and outputs a categorical distribution, as represented by three probability values associated with three curve types (see Figure 3a), while the curve feature predictor adopts a normal distribution output layer and outputs a normal distribution for each curve feature, as represented by its mean value *μ* and standard deviation *δ* (see Figure 3b).

Given the dual-distribution output at each molding condition, its expected stress–strain curve is ready to reconstruct, with an aleatoric uncertainty provided, as illustrated in Figure 3c. Note that, since the curve feature predictor would yield nonnull outputs for all curve features and cannot distinguish the curve type, a dominant curve type must be provided by the curve type classifier for stress–strain curve reconstruction. More technical details about the reconstruction rules are provided in the following section (see Section 3.3-(3)). Overall, the DNN architecture, combining the curve type classifier and curve feature regressor, forms a knowledge-informed neural network (KINN). This approach leverages the state-of-the-art simplicity of reduced hidden neurons for stress–strain curve prediction [1], with a total of 83 hidden neurons for the curve type classifier and 100 hidden neurons for the curve feature regressor, significantly reducing the data size requirement while maintaining model accuracy (see Section 3.3-(2)).

(2) Prediction Accuracy of the Dual-Distribution Neural Network

Now, we investigate the DNN model’s training performance and prediction accuracy. Regarding the DNN training process, the classifier and regressor are trained independently, and their loss functions are defined as the negative log-likelihood of true data under the predicted distribution. All training hyperparameters have been fine-tuned to optimize the model’s performance. The stochastic gradient descent (SGD) optimizer is adopted to optimize the neuron weights and biases in 2000 epochs, with an initial learning rate of 0.01 and a batch size of 6 curves per molding condition. We observed that the training loss rapidly decreases and plateaus at 0.4 for the classifier and 5.0 for the regressor. Additionally, the root mean square error (RMSE) and categorical accuracy (CA) reach 0.9 and 1, respectively, by the end of training (as shown in Figure S1). Note that, before training, all data are subjected to preprocessing to ensure the training performance, with the curve features and molding conditions subjected to standardization, and the curve types represented by one-hot representation. For null curve features in curve type I and II, the fracture point value is assigned to the null value as the regressor output. More details about the data preprocessing and the training process are provided in the Supplementary Materials.

**Figure 4:**
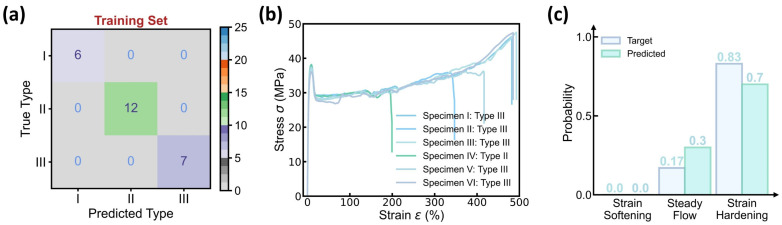
Prediction accuracy of the curve type classifier. (**a**) Confusion matrix of the training set. The dataset contains 27 molding conditions, wherein 25 conditions are selected as the training set, while the remaining 2 conditions serve as the test set. (**b**) Stress–strain curves in one test condition. (**c**) Predicted versus true categorical distribution of curve type in the test condition.

Figure 4 shows the prediction accuracy of the curve type classifier, wherein 25 molding conditions are selected as the training set and the remaining 2 conditions serve as the test set, and the confusion matrix of both the training and test sets exhibits a 100% classification accuracy. To validate the classifier’s performance, Figure 4b provides the stress–strain curves at one test molding condition, and the predicted versus true curve type distribution is provided in Figure 4c. Impressively, the model prediction assigns a 70% probability to curve type III and a 30% probability to curve type II, offering an excellent agreement with the experimental results of 83% type III and 17% type II. Considering the complexity of curve variations at each condition, the close match between predicted versus true distribution demonstrates the classifier’s capability in predicting the dominant curve type at each molding condition.

**Figure 5:**
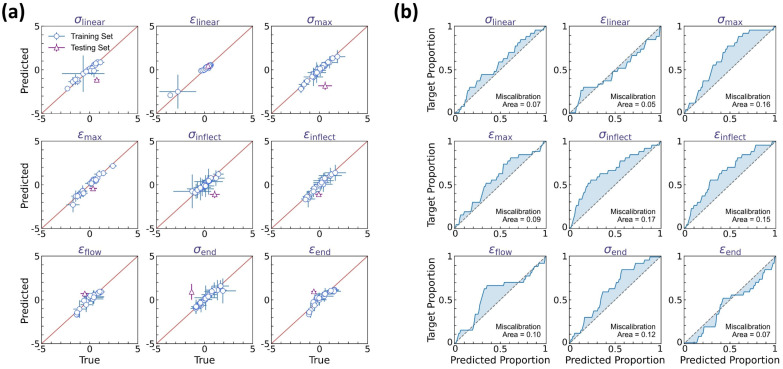
Prediction accuracy of the curve feature predictor. (**a**) Predicted versus true mean values for each curve feature, wherein the horizontal and vertical error bars represent the standard deviation of true versus predicted data, respectively. (**b**) Average calibration plot of observed versus predicted proportion in α-prediction interval for each curve feature, wherein α ranges from 0% to 100% to indicate the data proportion falling within the α-prediction interval.

Figure 5 shows the prediction accuracy of the curve feature regressor, wherein one molding condition is selected as the test set and the remaining 26 conditions are the training set. As the regressor outputs a normal distribution to quantify each curve feature’s aleatoric uncertainty, we adopt herein two types of *y* = *x* calibration curves to evaluate the prediction accuracy of the normal distribution’s mean *μ* (see Figure 5a) and standard deviation *δ* (see Figure 5b), respectively. Figure 5a shows the predicted versus true mean values for each curve feature, with the horizontal and vertical error bars representing the standard deviation of true versus predicted data, respectively. It is notable that all training data points are located around the *y* = *x* line, with the mean squared error (MSE) less than 0.15, which is considered satisfactory herein. More importantly, when extrapolating to the test condition, the regressor exhibits a reasonably good extrapolability to the test set—despite the training data size being extremely small and the test set being an extrapolation condition uncovered by the training condition regime.

Further, we evaluate the prediction accuracy of the normal distribution’s standard deviation *δ*, that is, the aleatoric uncertainty quantification at each molding condition. Figure 5b shows the average calibration plot of observed versus predicted data proportion in the *α*-prediction interval for each curve feature, wherein *α* ranges from 0% to 100% to indicate the data proportion falling within the *α*-prediction interval. Ideally, the predicted normal distribution is expected to satisfy the requirement that the observed proportion should be equal to *α* in the *α*-prediction interval, that is, forming a *y* = *x* line in the average calibration plot. Otherwise, the inconsistency between predicted versus true data distribution can be evaluated by the miscalibration area between the calibration curve and the *y* = *x* line, which identifies the distribution range deviating from the real data distribution, suggesting either insufficient or excessive uncertainty estimation. We find that, for each curve feature, the predicted normal distribution exhibits a satisfactory accuracy in describing the experimental data distribution, with an average miscalibration area of 0.11. And in most cases, these miscalibrations show a calibration curve above the *y* = *x* reference line (see Figure 5b), suggesting a slightly excessive uncertainty estimation, which is potentially beneficial for extrema estimation and reliability guidance thereof. Overall, these results demonstrate that the DNN model can accurately predict the dual-distribution representation of curve variation at each molding condition.

(3) Reconstructing Variational Curve from its Dual Distribution Representation

**Figure 6:**
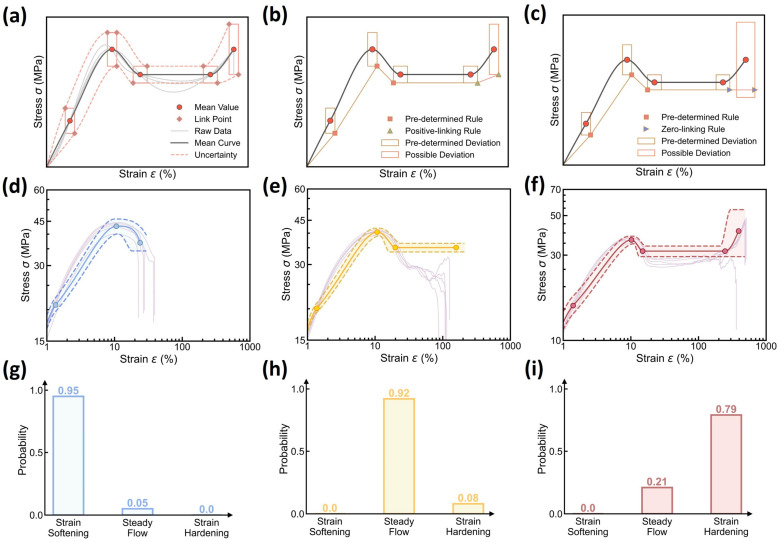
Stress–strain curve prediction using the DNN model. (**a**–**c**) Schematic illustrating the rules to reconstruct the expected stress–strain curve and its aleatoric uncertainty based on the dual distribution of curve types and features (see text for the details). (**d**–**f**) Examples of stress–strain curve prediction at different molding conditions, including (**d**) Tmold = 80 °C, Ppack = 80 MPa, Pinject = 30 MPa, and Rinject = 58.002 cm^3^/s; (**e**) Tmold = 50 °C, Ppack = 50 MPa, Pinject = 50 MPa, and Rinject = 58.002 cm^3^/s; and (**f**) Tmold = 20 °C, Ppack = 80 MPa, Pinject = 30 MPa, and Rinject = 41.43 cm^3^/s, wherein the shadow region represents the curve’s aleatoric uncertainty. Experimental data are also added as a reference. (**g**–**i**) Predicted curve type distributions at these molding conditions.

Based on the dual distribution prediction, we can reconstruct the expected stress–strain curve and its aleatoric uncertainty at each molding condition. Figure 6a–c illustrate the stress–strain curve reconstruction rules. First, given the dominant curve type, the expected mean curve is reconstructed by monotonic spline interpolation between the mean values of relevant curve features. Then in the same spirit, the aleatoric uncertainty of this reconstructed curve is provided by connecting the rectangle vertices associated with these curve feature points, wherein each rectangle represents the extrema boundary that encompasses the corresponding curve feature variation. Herein, the rectangle bounds are constructed using the 95% confidence intervals of curve feature distributions, that is, using μ±1.96×δ as the lower and upper bounds to maximally cover the variation range of curve feature points. Finally, considering the monotonic increase trend at the strain hardening regime, the connection between rectangle vertices generally follows the positive-slope linking rule (see Figure 6b), while in some scenarios with substantial fluctuation of the fracture point, the positive-slope linking rule may fail, and to rectify the systematic error, a zero-slope linking rule is applied instead, as shown in Figure 6c. Overall, the set of reconstruction rules offers a simple yet reliable approach to generate the expected stress–strain curve and its aleatoric uncertainty in an efficient manner.

(4) Predicting Curve Variation at Different Molding Conditions

Based on the reconstruction rules, we finally evaluate the stress–strain curve prediction of the DNN model at different molding conditions. Figure 6d–f showcase the prediction of the reconstructed stress–strain curve at different molding conditions and its aleatoric uncertainty, wherein the experimental curve data are added as a reference, and their predicted curve type distributions are provided in Figure 6g–i. Indeed, the reconstructed mean stress–strain curves offer an excellent agreement with their experimental curve references, and the established 95% confidence interval can apparently encompass all corresponding raw data curves within the predicted bounds. Further, to evaluate the model’s extrapolability to different molding conditions, we iteratively select from the dataset one molding condition as a test set, while the remaining 26 conditions serve as a training set, and it has been proved that the DNN model remains a reasonably good extrapolability to each test condition (see Supplementary Materials, Table S1). Despite some discrepancies between the predicted and experimental data, it can be concluded that the DNN model is capable of predicting stress–strain curve variation with a modest and reliable 95% confidence interval—that is, enabling aleatoric uncertainty quantification, ready for extrema estimation and reliability guidance after further incorporating the epistemic uncertainty (see Section 3.4).

### 3.4. Beyond Curve Variation: Uncertainty Quantification by Bayesian Neural Network

(1) Epistemic Uncertainty Induced by Model Deviation

**Figure 7:**
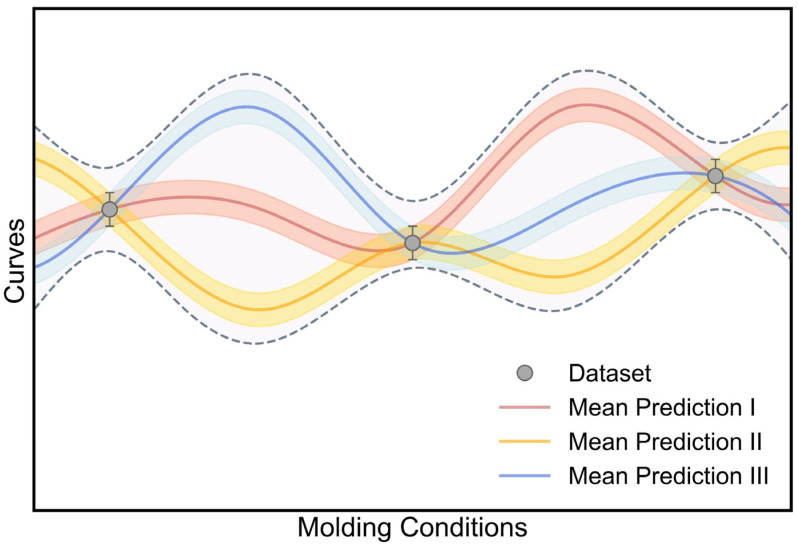
Schematic illustration of epistemic uncertainty induced by model deviation. The total uncertainty consists of aleatoric uncertainty (data deviation) and epistemic uncertainty (model deviation).

Relying on the distribution output layer, we have demonstrated that the DNN model can generate a probability density distribution to describe the experimental data distribution and quantify its aleatoric uncertainty. However, given the same experimental data, it is very likely that there exists a set of DNN models with different model parameters that can offer comparable model performance, as illustrated in Figure 7. In principle, these models can accurately describe the stress–strain curve variation at molding conditions within the experimental dataset but exhibit an evident model deviation between each other in the entire condition range (see Figure 7), which is ascribed to the uncertainty of model parameters—that is, “epistemic uncertainty” [17]. Here, relying on the Bayesian inference theorem (see Section 3.4-(2)), the Bayesian neural network (BNN) approach would be applied to quantify the epistemic uncertainty of the DNN model [21,30,31]. We expect that, by incorporating the epistemic uncertainty, the model prediction would account for not only the data deviation but also the model deviation (see Figure 7), so as to enhance the prediction accuracy and reliability.

(2) Bayesian Inference Theorem

In the provided dataset $D=\left\{x,y\right\}={\left\{{x_{i},y}_{i}\right\}}_{i=1:n}$, where x represents input samples and y represents output samples, the ML model is trained to produce results through the adjustment of model parameters $\omega \in R^{d}$, where d denotes the number of model parameters (i.e., neuron weights and biases) is determined by the number of neurons in the DNN model herein. By minimizing the loss function $L\left(f^{\omega }\left(x_{i}\right),y_{i}\right)\;$ between the model $f^{\omega }\left(x_{i}\right)\;$ and the target value $y_{i}$, traditional ML models typically seek a specific set of parameters ω to establish a one-to-one mapping relationship between input samples x and output samples y.

In contrast, Bayesian methods, represented by Equation (1), provide a unique capability to capture both the aleatoric and epistemic uncertainty by stochastic probability models [21,22]:

(1) $$p\left(y|x,\omega \right)=N\left(y;f^{\omega }\left(x\right),\delta_{n}^{2}\right)\;\;$$

wherein $p\left(\cdot \right)$ is the probability density function (PDF), herein modeled as a normal distribution with a mean of $f^{\omega }\left(x\right)$ and a variance of ${\delta_{n}^{2}}^{\omega }\left(x\right)$ to quantify aleatoric uncertainty, serving as the prior. It is assumed that the data in the dataset D are independent of each other, allowing the likelihood $p\left(D|\omega \right)$ to be expressed as Equation (2):

(2) $$p\left(D|\omega \right)=\prod_{i=1}^{n}N\left(y_{i};f^{\omega }\left(x_{i}\right),\delta_{n}^{2}\right)$$

and according to Bayes’ theorem [30,31], the posterior PDF $p\left(\omega |D\right)$ can be computed by Equation (3), which is intrinsically connected to the representation of epistemic uncertainty:

(3) $$p\left(\omega |D\right)=\frac{p\left(D|\omega \right)p_{0}\left(\omega \right)}{p\left(D\right)}\;\;\;\;$$

wherein a prior PDF $p_{0}\left(\omega \right)$ is set as the independent Gaussian for each weight and bias in BNN neurons, collectively describing the possible distribution of $f^{\omega }\left(x\right)$ and ${\delta_{n}^{2}}^{\omega }\left(x\right)$. Then, given the new input $x^{*}$, the predicted output distribution $y^{*}$ is defined by Equation (4) as follows:

(4) $$p\left(y^{*}|x^{*},D\right)=\int p\left(y^{*}|x^{*},\omega \right)p\left(\omega |D\right)d\omega \;\;$$

which represents the total uncertainty consisting of both the aleatoric and epistemic uncertainty. Employing variational inference (VI) to approximate the true posterior $p\left(\omega |D\right)$ via the BNN model [31], a factorized Gaussian distribution $q_{\theta }\left(\omega \right)$ is utilized, where the BNN model parameters $\theta ={\left\{{\mu_{i},\delta }_{i}\right\}}_{i=1:d}$ comprises the mean and standard deviation in each independent Gaussian to describe the distribution of each neuron weight or bias, as expressed by Equation (5):

(5) $$q_{\theta }\left(\omega \right)=\prod_{i=1}^{d}N\left(\omega_{i};\mu_{i},\delta_{i}\right)$$

By substituting $p\left(\omega |D\right)$ in Equation (4) with $q_{\theta }\left(\omega \right)$, the total uncertainty $p\left(y^{*}|x^{*},D\right)$ is derived as Equation (6):

(6) $$p\left(y^{*}|x^{*},D\right)=\int p\left(y^{*}|x^{*},\omega \right)q_{\theta }\left(\omega \right)d\omega \;\;\;\;$$

**Figure 8:**
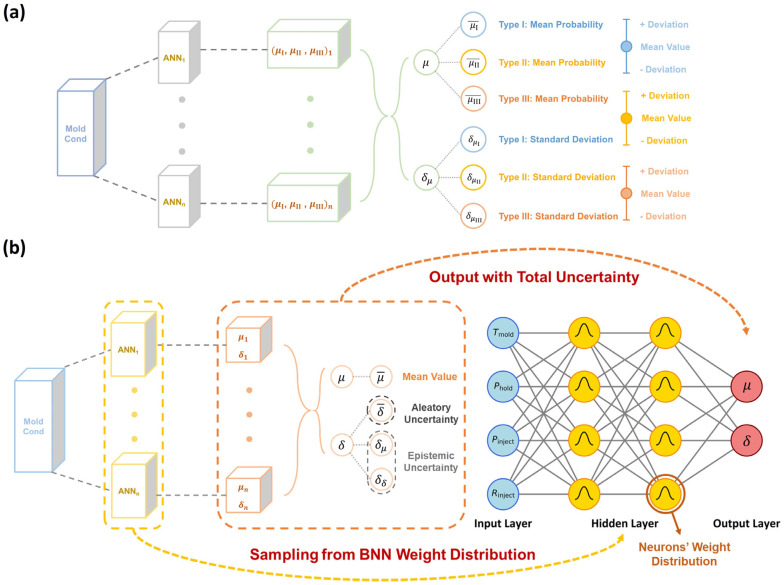
Uncertainty quantification by dual-distribution Bayesian network (DBN). (**a**) Schematic illustrating the working principle of a curve-type classifier using a Bayesian neural network (BNN), wherein the weights and biases in BNN neurons are sampled from their independent Gaussian distributions so that the BNN-based classifier is equivalent to multiple classifiers based on artificial neural networks (ANNs) under different settings of weights and biases. By statistical averaging, the mean *μ* and standard deviation *δ* of curve type probability are obtained. (**b**) BNN-based curve feature regressor, wherein the multiple mean *μ* and standard deviation *δ* of curve feature distribution are statistically averaged to obtain the expected mean $\bar{\mu }$ and aleatoric uncertainty $\bar{\delta }$, and meanwhile, the standard deviation of *μ* and *δ* are computed as the epistemic uncertainty $\delta_{\mu }$ and $\delta_{\delta }.$

As a result, the mean $E\left[y^{*}|x^{*},D\right]$ and the upper-bound variance $Var\left[y^{*}|x^{*},D\right]$ are computed by Equations (7) and (8), respectively [21,31]:

(7) $$E\left[y^{*}|x^{*},D\right]=E_{q_{\theta }\left(\omega \right)}\left[E\left[y^{*}|x^{*},\omega \right]\right]=E_{q_{\theta }\left(\omega \right)}\left[f^{\omega }\left(x^{*}\right)\right]$$

(8) $$Var\left[y^{*}|x^{*},D\right]=E_{q_{\theta }\left(\omega \right)}\left[Var\left(y^{*}|x^{*},\omega \right)\right]+Var_{q_{\theta }\left(\omega \right)}\left(E\left[y^{*}|x^{*},\omega \right]\right)+Var_{q_{\theta }\left(\omega \right)}\left(Var\left(y^{*}|x^{*},\omega \right)\right)=\underset{Aleatoric\;Uncertainty}{\underbrace{E_{q_{\theta }\left(\omega \right)}\left[{\delta_{n}^{2}}^{\omega }\left(x^{*}\right)\right]}}+\underset{Epistemic\;Uncertainty}{\underbrace{Var_{q_{\theta }\left(\omega \right)}\left(f^{\omega }\left(x^{*}\right)\right)+Var_{q_{\theta }\left(\omega \right)}\left({\delta_{n}^{2}}^{\omega }\left(x^{*}\right)\right)}}$$

wherein the expected mean $E_{q_{\theta }\left(\omega \right)}\left[f^{\omega }\left(x^{*}\right)\right]$ (referred to as $\bar{\mu }$ below) is calculated via sampling neuron weights and biases from their Gaussian distributions in the well-trained BNN model (see Figure 8). Accordingly, the aleatoric uncertainty $E_{q_{\theta }\left(\omega \right)}\left[\delta_{n}^{2}\left(x^{*}\right)\right]$ (referred to as $\bar{\delta }$ below) and the epistemic uncertainty $Var_{q_{\theta }\left(\omega \right)}\left(f^{\omega }\left(x^{*}\right)\right)$ and $Var_{q_{\theta }\left(\omega \right)}\left({\delta_{n}^{2}}^{\omega }\left(x^{*}\right)\right)$ (referred to as $\delta_{\mu }$ and $\delta_{\delta }$, respectively, below) can be obtained to evaluate the deviation of data and model separately (see Section 3.4-(3)).

(3) Total Uncertainty Quantification by a Dual-Distribution Bayesian Network

According to the above BNN approach, we construct herein a dual-distribution Bayesian network (DBN) to simultaneously address both the aleatoric and epistemic uncertainty. Compared to the DNN model, the DBN model simply replaces all hidden neurons with the BNN neuron type. Unlike setting a single value for weights and biases in traditional neurons, the BNN neurons use some distribution parameters to describe the distribution of weights and biases (i.e., mean and standard deviation in Gaussian distribution herein), and these distribution parameters are optimized via the same training scheme as the DNN model. After training, we randomly sample from the distributions of weights and biases to output multiple dual-distribution results so that the DBN model is equivalent to multiple DNN models under different settings of weights and biases. By statistically analyzing the multiple outputs based on Equation (8), the DBN model enables quantification of both the aleatoric and epistemic uncertainty.

Figure 8 illustrates the quantification of epistemic uncertainty using the DBN model, wherein the weights and biases in BNN neurons are sampled from their independent Gaussian distributions so that the BNN-based curve type classifier and curve feature regressor are equivalent to multiple classifiers and regressors based on artificial neural networks (ANNs) under different settings of weights and biases. By statistical averaging, the BNN-based classifier outputs the mean probability for each curve type (denoted as $\bar{\mu_{i}}$), along with a standard deviation ($\delta_{\mu_{l}}$) that encapsulates the potential deviation in curve type classification, that is, the epistemic uncertainty of the classifier model (see Figure 8a). Similarly, the BNN-based regressor yields multiple mean *μ* and standard deviation *δ* for each curve feature distribution and then statistically averages the multiple values to obtain the expected mean $\bar{\mu }$ and aleatoric uncertainty $\bar{\delta }$ for each curve feature, along with the standard deviation of *μ* and *δ* (denoted as $\delta_{\mu }$ and $\delta_{\delta }$) computed as the epistemic uncertainty of the regressor model (see Figure 8b). Overall, by introducing a BNN hidden neuron into the DNN architecture, the DBN model can not only inherit all the DNN model’s attributes for stress–strain curve prediction but also quantify the epistemic uncertainty of the DNN model toward enhanced model reliability.

### 3.5. Curve Uncertainty Prediction by the Dual-Distribution Bayesian Model

(1) Prediction Accuracy of the Dual-Distribution Uncertainty

We now evaluate the DBN model’s prediction accuracy by taking into consideration the epistemic uncertainty of its dual distribution output. Note that the DBN and DNN model shares the same training scheme, and the training details are provided in Supplementary Materials. Unlike the DNN model that predicts only one dual-distribution output at a molding condition, the DBN model can generate multiple dual-distribution outputs at the same molding condition, for example, 100 outputs herein, via sampling different combinations of weights and biases from their independent distributions in BNN neurons. The final output of the DBN model is the “dual-distribution uncertainty”—that is, the mean and variance of the multiple dual-distribution outputs (see Figure 8), which represent, respectively, the aleatoric and epistemic uncertainty in predicting a stress–strain curve at the molding condition.

**Figure 9:**
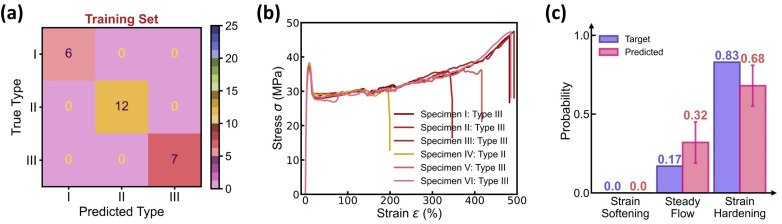
Prediction accuracy of the BNN-based curve type classifier. (**a**) Confusion matrix of the training set. The BNN-based classifier uses the same training scheme as the DNN model (see Figure 4). (**b**) Stress–strain curves in one test condition. (**c**) Predicted versus true categorical distribution of curve type in the test condition. The classifier predicts a mean probability with an error bar provided for each curve feature.

Figure 9 shows the prediction accuracy of the BNN-based curve type classifier in the DBN model, wherein the dominant curve type at each molding condition is determined by averaging over 100 predictions of curve type distribution, and the confusion matrix of both the training and test sets exhibits a 100% classification accuracy. Similar to the classifier performance in the DNN model (see Figure 4), the BNN-based classifier offers a curve type distribution in excellent agreement with its experimental data reference (see Figure 9b,c). Moreover, the predicted distribution contains not only the mean probability for each curve type but also the standard deviation that quantifies the classifier’s epistemic uncertainty, thus fundamentally enhancing the model prediction reliability.

**Figure 10:**
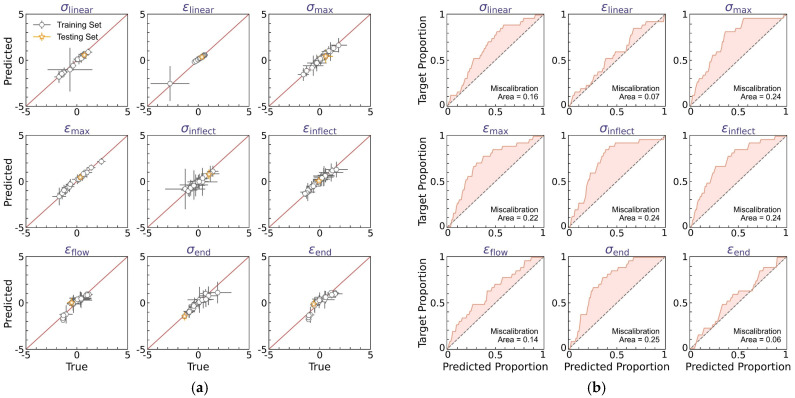
Prediction accuracy of the BNN-based curve feature predictor. (**a**) Predicted versus true mean values for each curve feature, wherein the horizontal and vertical error bars represent the variance of true versus predicted data, respectively. The predicted variance is the upper-bound variance computed by Equation (8). (**b**) Average calibration plot of observed versus predicted proportion in *α*-prediction interval for each curve feature, wherein *α* ranges from 0% to 100% to indicate the data proportion falling within the *α*-prediction interval.

Figure 10 shows the prediction accuracy of the BNN-based curve feature regressor in the DBN model, wherein two types of *y* = *x* calibration curves are provided to evaluate the accuracy of, respectively, the predicted mean *μ* (see Figure 10a) and variance *δ* (see Figure 10b) of the experimental curve feature distribution. Note that the mean and variance are determined by averaging over 100 predictions of curve feature distribution, as computed by Equations (7) and (8), respectively, and herein, the upper-bound variance is utilized to benefit extrema estimation by combining all sources of uncertainty, that is, a variance of $\bar{\delta }+\delta_{\mu }+\delta_{\delta }$ (see Equation (8)). Figure 10a shows the predicted versus true mean values for each curve feature, with the horizontal and vertical error bars representing the variance of true versus predicted data, respectively, and the average calibration plot of observed versus predicted data proportion in the *α*-prediction interval is provided in Figure 10b.

Similar to the regressor performance in the DNN model (see Figure 5), the BNN-based regressor offers a satisfactory match between predicted versus true mean values, with the training set MSE less than 0.15, comparable to that of the DNN model (i.e., an MSE of 0.15, see Figure 5a). And when extrapolating to the test set, the regressor exhibits evidently better extrapolability than the DNN model (see Figure 10a), suggesting that the statistical average operation of BNN-based outputs is likely to eliminate the bias effect of model deviation and, therefore, enhance the model’s extrapolability. We then evaluate the predicted upper-bound variance based on the miscalibration area in the average calibration plot for each curve feature (see Figure 10b). As expected, by considering the upper-bound variance, the predicted normal distribution exhibits an expanded width yet remains satisfactory in describing the experimental data distribution, with an average miscalibration area of 0.18, higher than that of the DNN model (i.e., an area of 0.11, see Figure 5b), and to a more evident extent, these miscalibrations show a calibration curve above the *y* = *x* reference line (see Figure 10b), suggesting an excessive yet modest uncertainty estimation, which is considered to be beneficial herein for extrema estimation.

(2) Reconstructing Curve Uncertainty from its Dual Distribution Uncertainty

**Figure 11:**
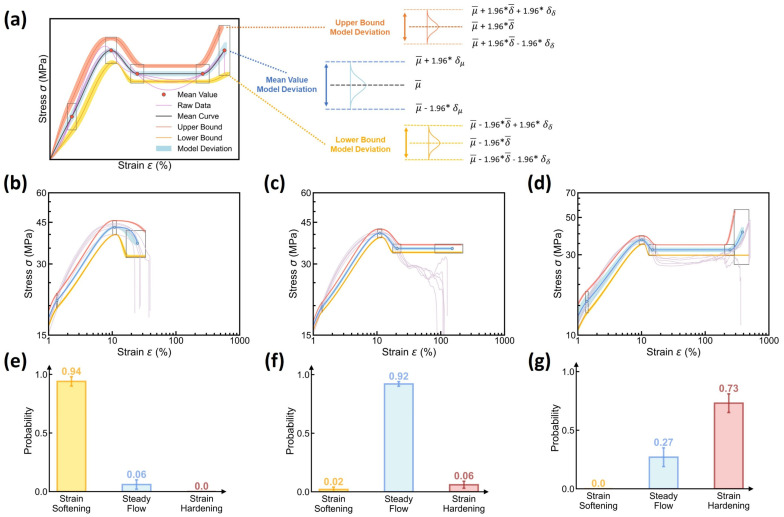
Reconstruction of the stress–strain curve and its uncertainty using the DBN model. (**a**) Schematic illustrating the rules to reconstruct the expected stress–strain curve and its aleatoric and epistemic uncertainty based on the mean and variance of multiple dual-distribution outputs (see text for the details). (**b**–**d**) Examples of the reconstructed stress–strain curve and its aleatoric and epistemic uncertainty at different molding conditions, including (**d**) Tmold = 80 °C, Ppack = 80 MPa, Pinject = 30 MPa, and Rinject = 58.002 cm^3^/s; (**e**) Tmold = 50 °C, Ppack = 50 MPa, Pinject = 50 MPa, and Rinject = 58.002 cm^3^/s; and (**f**) Tmold = 20 °C, Ppack = 80 MPa, Pinject = 30 MPa, and Rinject = 41.43 cm^3^/s, wherein the shadow regions represent the epistemic uncertainty. Experimental data are also added as a reference. (**e**–**g**) Predicted curve type distributions at these molding conditions.

Based on the dual-distribution output of DBN model, we can reconstruct the expected stress–strain curve and both its aleatoric and epistemic uncertainty, following the same reconstruction rules applied to the DNN model (see Figure 6). Figure 11 shows the reconstruction of the stress–strain curve and its uncertainty based on the DBN model. Compared to DNN-based reconstruction, DBN-based reconstruction provides a statistically model-averaged estimation of the expected mean $\bar{\mu }$ and variance $\bar{\delta }$ of curve variation at each molding condition (see Equations (7) and (8)), fundamentally eliminating the biased-model effect on aleatoric uncertainty quantification. More distinctively, the reconstruction approach introduces two new sources of uncertainty, that is, the epistemic uncertainty of μ and δ—herein denoted as $\delta_{\mu }$ and $\delta_{\delta }$ (see Equation (8))—which are reconstructed as three new uncertainty regions associated to the stress–strain curve’s mean value, lower bound, and upper bound, respectively (see Figure 11a). For simplicity, each epistemic uncertainty region is approximated by a normal distribution at a 95% confidence interval, given the computed mean ($\bar{\mu }$ and $\bar{\delta }$) and variance ($\delta_{\mu }$ and $\delta_{\delta }$). Based on these reconstruction rules, the visualization of the stress–strain curve prediction distinctively differentiates between the aleatoric and epistemic uncertainty.

Figure 11b–d showcase the reconstructed stress–strain curve and its aleatoric and epistemic uncertainty at different molding conditions, wherein the experimental curve data are added as a reference, and their predicted curve type distributions are provided in Figure 11e–g. Unlike the DNN model solely characterizing the aleatoric uncertainty (see Figure 6), the DBN model provides a reconstructed stress–strain curve with a taxonomy of different sources of curve uncertainty at each molding condition. Notably, by incorporating epistemic uncertainty, these curve reconstructions remain closely similar to DNN-based reconstructions, revealing that the epistemic uncertainty is controlled within a modest extent at a small data size. When extrapolating to different test molding conditions, it is notable that the DBN model can generally offer modest epistemic uncertainty at a magnitude negligible or comparable to the aleatoric uncertainty (see Supplementary Materials), demonstrating the reliability of DBN model in predicting stress–strain curve at a modest uncertainty using small-size dataset, with both the epistemic and aleatoric uncertainty differentiated and visualized.

(3) Curve Uncertainty Quantification at Different Molding Conditions

**Figure 12:**
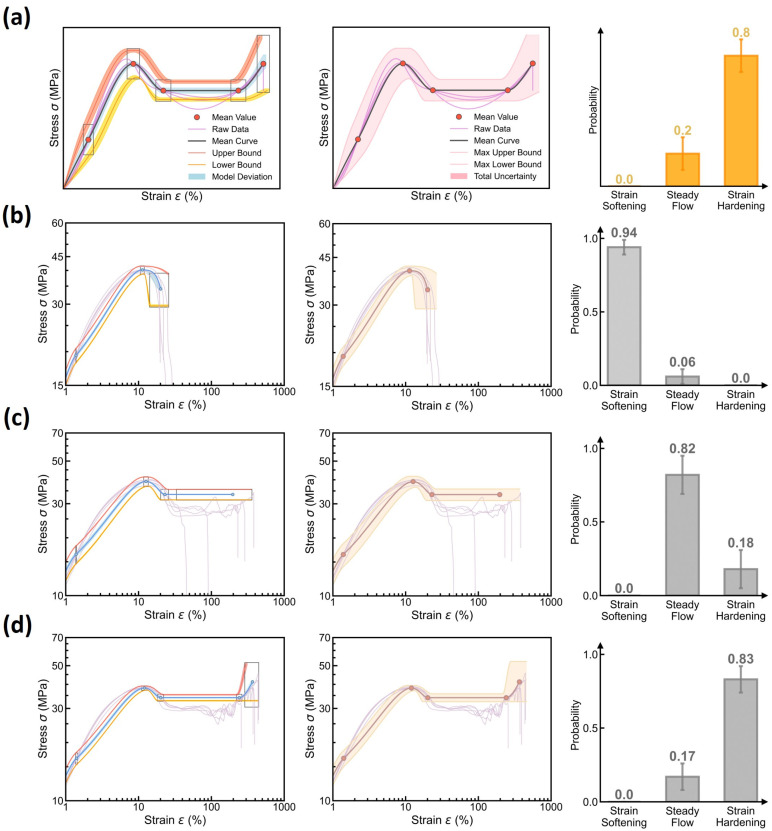
Maximum uncertainty quantification of stress–strain curve prediction using the DBN model. (**a**) Schematic illustrating the maximum uncertainty bounds (middle panel) attained by summing up all uncertainty sources (left panel), that is, a variance of $\bar{\delta }+\delta_{\mu }+\delta_{\delta }$ (see Equation (8)) for a normal distribution at a 95% confidence interval. The predicted curve type distribution is provided in the right panel. (**b**–**d**) Examples of maximum uncertainty quantification at different molding conditions, including (**b**) *T*_mold_ = 80 °C, *P*_pack_ = 20 MPa, *P*_inject_ = 50 MPa, and *R*_inject_ = 58.002 cm^3^/s and (**c**) *T*_mold_ = 50 °C, *P*_pack_ = 20 MPa, *P*_inject_ = 70 MPa, and *R*_inject_ = 41.43 cm^3^/s and (**d**) *T*_mold_ = 20 °C, *P*_pack_ = 50 MPa, *P*_inject_ = 70 MPa, and *R*_inject_ = 41.43 cm^3^/s.

Based on the visualized uncertainty sources, we finally establish the maximum uncertainty quantification of the stress–strain curve prediction. According to the upper-bound variance estimation in curve uncertainty (see Equation (8)), the maximum uncertainty can be attained by summing up all uncertainty sources, that is, a normal distribution with a mean $\bar{\mu }$ and variance $\bar{\delta }+\delta_{\mu }+\delta_{\delta }$ at 95% confidence interval, as illustrated in Figure 12a. Figure 12b–d show some examples of the maximum uncertainty quantification at different molding conditions (see the middle panel), with the uncertainty source visualization and the predicted curve type distribution provided in the left and right panels, respectively. Indeed, we find that the maximum uncertainty boundary can properly encompass its experimental stress–strain curves at each molding condition, especially for those key feature points captured by the curve feature regressor. Note that, despite their satisfactory alignment, the predictions and experimental data exhibit some discernible discrepancies, which deserve future investigations and might be rooted in (i) the predefined normal distribution of curve features and (ii) the monotonic spline interpolation in curve reconstruction rules. Importantly, by summing up all uncertainty sources, we find that the maximum uncertainty remains a modest range slightly larger than its raw data distribution, which echoes the constrained epistemic uncertainty of the DBN model, and these findings remain true when extrapolating to different test molding conditions (see Supplementary Materials). Based on the modest maximum-uncertainty, it concludes that the DBN model is adept at extrema estimation and reliability guidance, that is, “predict stress–strain curve with confidence”.

## 4. Conclusions

Overall, this study pioneers a machine learning framework—the dual Bayesian network (DBN) model—to predict complex stress–strain curves with reliable and modest uncertainty, even with a small dataset. The model excels in differentiating between aleatoric and epistemic uncertainty and visualizing all sources of uncertainty. Notably, by accounting for all uncertainties, the maximum uncertainty of each prediction is controlled within a modest range, effectively encompassing the experimental data, despite the challenging task of extrapolating across the entire design space with limited data points. This balance between data minimization and uncertainty quantification is achieved through the following two key strategies of the DBN model: (i) the state-of-the-art simplicity of the dual classifier–regressor architecture and (ii) the small yet high-quality dataset generated via the Taguchi sampling method. We believe the DBN model’s capabilities are potentially transferable to a variety of curve-type properties, making it versatile for extrema estimation and reliability analysis in material property predictions.

## Data Availability

The original contributions presented in this study are included in the article and Supplementary Material. Further inquiries can be directed to the corresponding author.

## Supplementary Materials

The following supporting information can be downloaded at: https://www.mdpi.com/article/10.3390/polym17040550/s1, Figure S1: Training performance of DBN model. (a) Training loss of curve feature regressor as a function of training epochs. The loss function is defined as negative log-likelihood loss. (b) Training loss of curve type classifier. (c) Root mean square error (RMSE) of curve feature regressor as a function of training epochs for the training set. (d) Categorical accuracy (CA) of curve type classifier as a function of training epochs for the training set. (e) Evolution of regressor RMSE as a function of training epochs for the validation set. (f) Evolution of classifier CA as a function of training epochs for the validation set; Table S1: List of DBN model predictions at each of the 27 molding conditions. For each test condition in the first column, the model is trained with the other 26 conditions. The second column presents the result of curve type classifier with error bar indicating its uncertainty and the third column is the curve reconstruction output with one plot indicating all uncertainty sources (upper panel) and one offering the maximum uncertainty by summing up all uncertainties (lower panel); Table S2: List of 27 molding conditions used for prediction. References [32,33,34,35] are cited in the Supplementary Materials.
