# Supplementary material for: Predicting Stress–Strain Curve with Confidence: Balance Between Data Minimization and Uncertainty Quantification by a Dual Bayesian Model
Source: Polymers (Basel). 2025 Feb 19;17(4):550. doi: 10.3390/polym17040550 (PMC11860118; doi:10.3390/polym17040550)
Supplement: Supplementary file 1 [file polymers-17-00550-s001.zip › polymers-3399420-supplementary.pdf]

polymers

MDPI

Supplementary Materials

Predicting Stress–Strain Curve with Confidence: Balance Between Data Minimization and Uncertainty Quantification by a Dual Bayesian Model

Tianyi Li [1,2], Zhengyuan Chen [1,2], Zhen Zhang [3], Zhenhua Wei [4], Gan-Ji Zhong [2,5], Zhong-Ming Li [2,5,*] and Han Liu [1,2,5,*]

[1] SOlids inFormaTics AI-Laboratory (SOFT-AI-Lab), Sichuan University, Chengdu 610065, China; tianyili.dave@outlook.com (T.L.); 13258157129@163.com (Z.C.)
[2] College of Polymer Science and Engineering, Sichuan University, Chengdu 610065, China; ganji.zhong@scu.edu.cn
[3] College of Mathematics and Physics, Chengdu University of Technology, Chengdu 610059, China; zhen.zhang@cdut.edu.cn
[4] Department of Ocean Science and Engineering, Southern University of Science and Technology, Shenzhen 518055, China; weizh@sustech.edu.cn
[5] State Key Laboratory for Polymer Materials Engineering, Sichuan University, Chengdu 610065, China
* Correspondence: zmli@scu.edu.cn (Z.-M.L.); happylife@ucla.edu (H.L.)

S1. Data Preprocessing

Despite the extraction of feature data from experimental datasets and efforts to reduce computational complexity, directly inputting unprocessed data remains suboptimal due to significant scaling differences that can markedly affect final outcomes [1]. To address this issue, data transformation techniques have been employed to standardize the scale of the data without altering their inherent correlations.

In this study, substantial deviations between experimental data are observed (see Figure 2c). To mitigate the impact of these deviations, a sequential transformation process is applied: first, a natural logarithmic transformation to diminish biases caused by outliers, and second, standardization to ensure a homogeneous distribution across different features. This dual transformation approach is implemented to optimize model performance [2,3].

The effectiveness of this approach is demonstrated through predictive performance improvements. Initially, the dataset (Data I) is prepared using the aforementioned transformations to train the neural network, effectively minimizing outlier effects. Subsequently, a secondary dataset (Data II), which is standardized but not logarithmically transformed, is introduced for prediction purposes. Due to the standardization process, Data I and Data II exhibited similar distributions. This methodology allows the model to converge more effectively, as the training phase have already accounted for and eliminated outlier effects.

S2. Model Accuracy Calibration

To gauge the DBN model's training performance and prediction accuracy, mathematical calibration tools are employed, including negative log-likelihood loss, root mean square error, and categorical accuracy [4]. In this study, negative log-likelihood is applied to both the curve feature regressor and the curve type classifier as the loss function during

 https://doi.org/10.3390/polym17040550

training process. Root mean square error (RMSE) is utilized to evaluate the regressor performance, while categorical accuracy (CA) is employed to evaluate the classifier performance. These calibration metrics play a critical role in estimating the precision and reliability of the model's predictions.

S2.1 Training Accuracy of Curve Feature Predictor

The negative log-likelihood loss plays a crucial role in the training process by aligning the predicted distribution with the true values to minimize discrepancies [4,5]. In the case of the predictor's loss, illustrated in Figure S1a, the epoch is set at 2000. Initially, the loss stands at 30, significantly decreasing to below 10 after 500 epochs and eventually stabilizing around 5 by the 2000th epoch.

Moving to root mean square error (RMSE), particularly vital in regression scenarios, it measures the average magnitude of errors between predicted and actual values. This metric is computed as the square root of the average of squared differences between predicted and true values.

Examining the RMSE trends with the increase in epochs, the training set's RMSE fluctuates around 1 after 1000 epochs, signifying convergence (see Figure S1c). Similarly, for the testing set, a convergent trend is observed after 1500 epochs (see Figure S1e). Despite some fluctuation, especially in the cross-validated testing set, which hovers around 0.9, the overall trend indicates model convergence and precise predictive capabilities.

S2.2 Training Accuracy of Curve Type Classifier

For the classifier employing the one-hot format to predict curve types, the training process involves 10,000 epochs. Despite more intense data fluctuations, the negative log-likelihood loss plateaus around the 500th epoch, stabilizing at approximately 0.4 (see Figure S1b). This plateau indicates the training convergence.

In the realm of classification problems, categorical accuracy (CA) is a key metric to gauges the proportion of correctly predicted observations among the total observations. For the training set, CA reaches 90% by the 2,000th epoch (see Figure S1d), and this classifier saves the optimal neuron weights when CA reaches 100%. In the validation results, despite observing significant fluctuations, randomly selected data points demonstrate the potential for CA to reach 100%, thereby providing accurate predictions (see Figure S1f).

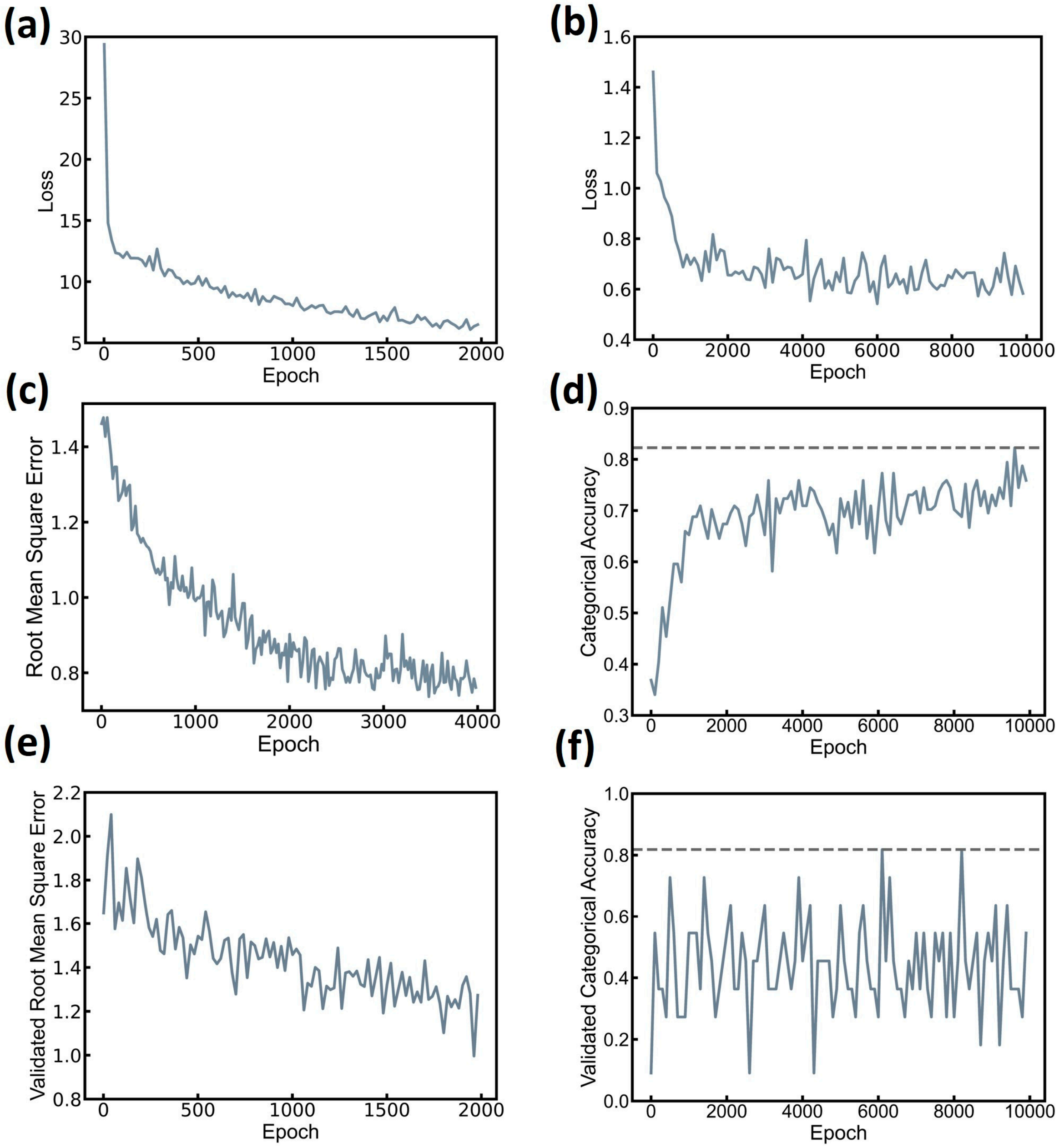

Figure S1. Training performance of DBN model. (**a**) Training loss of curve feature regressor as a function of training epochs. The loss function is defined as negative log-likelihood loss. (**b**) Training loss of curve type classifier. (**c**) Root mean square error (RMSE) of curve feature regressor as a function of training epochs for the training set. (**d**) Categorical accuracy (CA) of curve type classifier as a function of training epochs for the training set. (**e**) Evolution of regressor RMSE as a function of training epochs for the validation set. (**f**) Evolution of classifier CA as a function of training epochs for the validation set.

S3. Validation of Model Extrapolability at Various Test Set

Table S1 lists the DBN model's extrapolation results at various test set, where each molding condition listed in the first column represents the test set, while the remaining 26 samples serve as training set. Based on the trained dual neural network, the predicted curve type distribution for the test set is presented in the second column, while the reconstructed stress-strain curve prediction with all uncertainty sources (see upper panel) and the maximum uncertainty (see lower panel) is presented in the third column.

The DBN model enables precise predictions for all test samples. Even in challenging cases, such as sample 13, which originally had 50% of experimental results as type II and the other 50% as type III, the classifier reasonably infers and concludes that the curve is more likely to be type III. For the predictor, almost all reconstructed curves closely matched the experimental results, particularly in the maximum uncertainty plot. When extrapolating to unknown design space, the model can extrapolate to cover the majority of experimental curves, ensuring the model provides 95% confidence, as demonstrated by sample 1. Overall, these extrapolation validations confirm that the DBN model can deliver accurate predictions for extrema estimation and reliability guidance.

The detailed molding conditions are also listed in Table S2.

Table S1. List of DBN model predictions at each of the 27 molding conditions. For each test condition in the first column, the model is trained with the other 26 conditions. The second column presents the result of curve type classifier with error bar indicating its uncertainty and the third column is the curve reconstruction output with one plot indicating all uncertainty sources (upper panel) and one offering the maximum uncertainty by summing up all uncertainties (lower panel).

<table>
<tr><th>Test Molding Parameter: $\{\boldsymbol{P}_{\textbf{inject}}, \boldsymbol{R}_{\textbf{inject}}, \boldsymbol{P}_{\textbf{pack}}, \boldsymbol{T}_{\textbf{mold}}\}$</th><th>Curve Type Classifier</th><th>Curve Feature Predictor</th></tr>
<tr><td rowspan="2">Sample 1:
[20, 20, 30, 24.858]</td><td rowspan="2">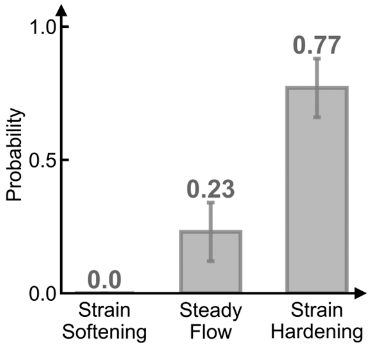
</td><td>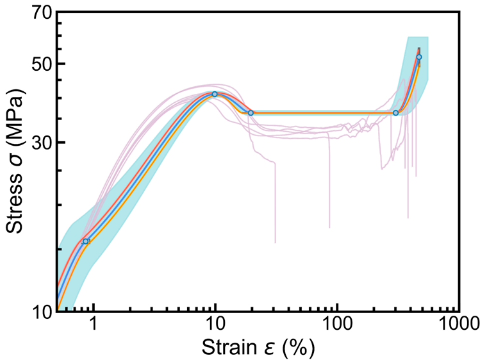
</td></tr>
<tr><td>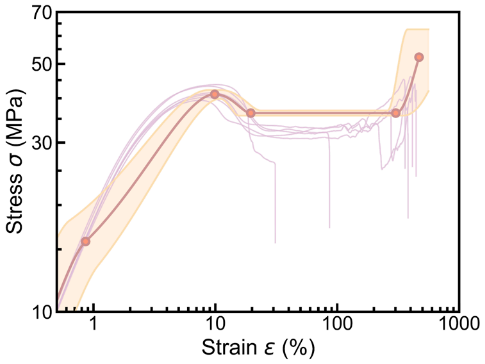
</td></tr>
</table>

Sample 2:

[20, 50, 50, 24.858]

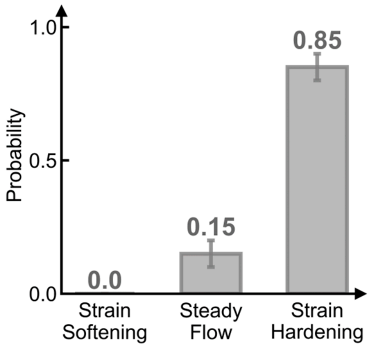

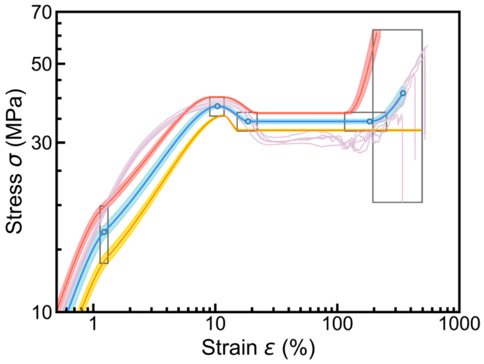

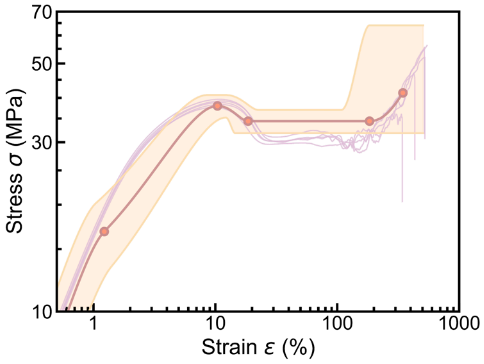

Sample 3:

[20, 80, 70, 24.858]

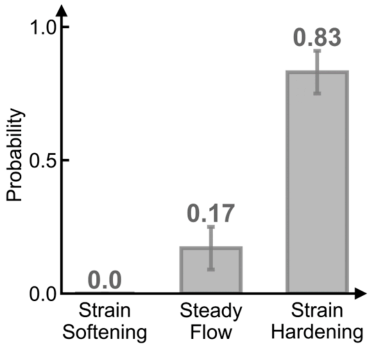

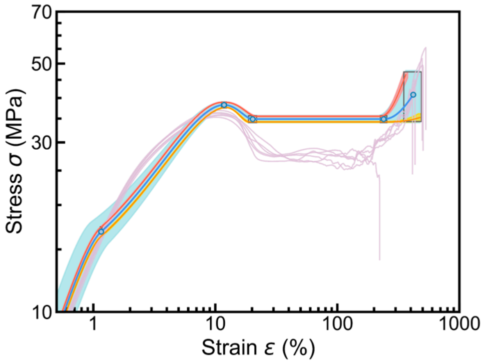

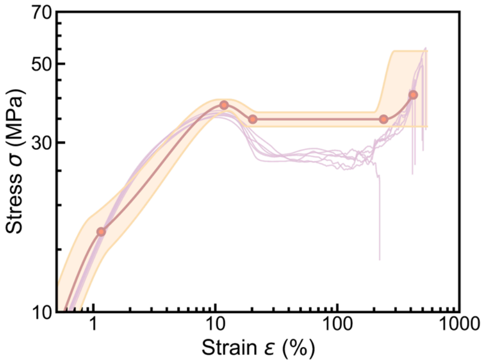

Sample 4:

[50, 20, 50, 24.858]

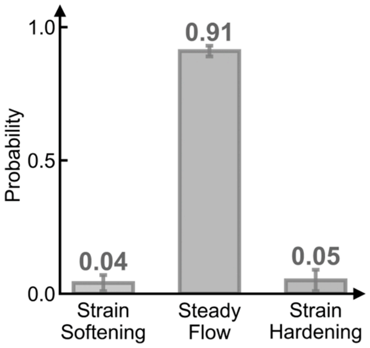

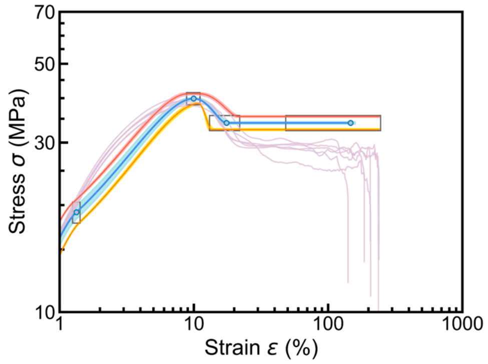

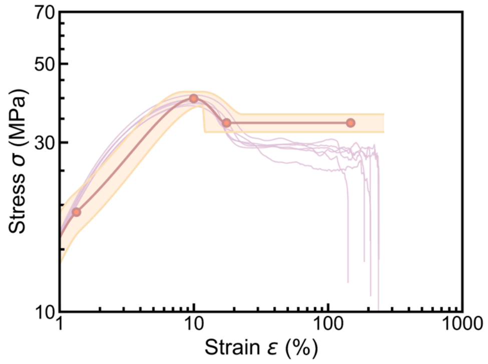

Sample 5:

[50, 50, 70, 24.858]

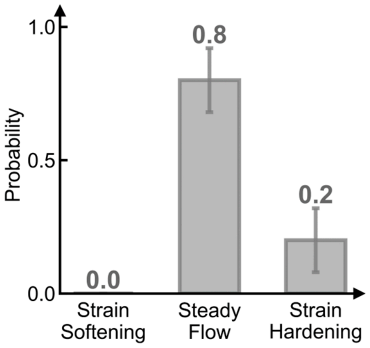

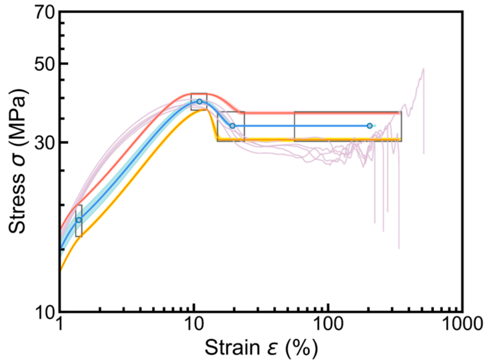

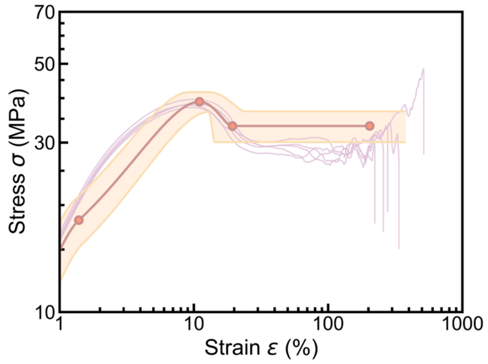

Sample 6:

[50, 80, 30, 24.858]

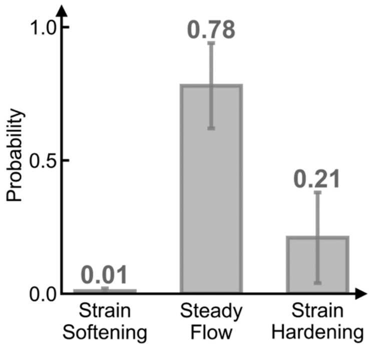

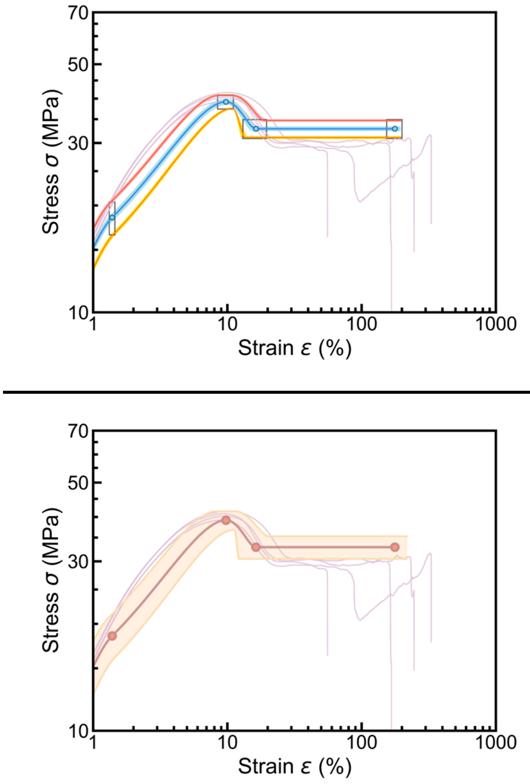

Sample 7:

[80, 20, 70, 24.858]

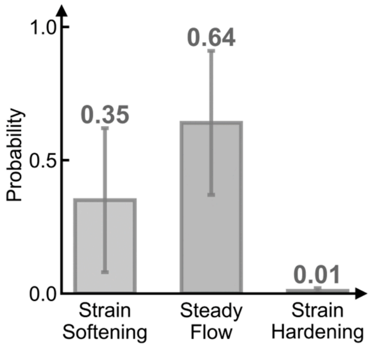

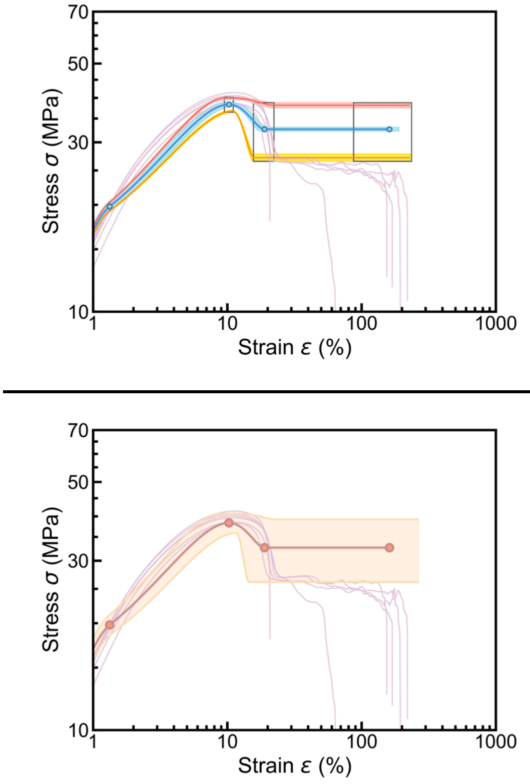

Sample 8:

[80, 50, 30, 24.858]

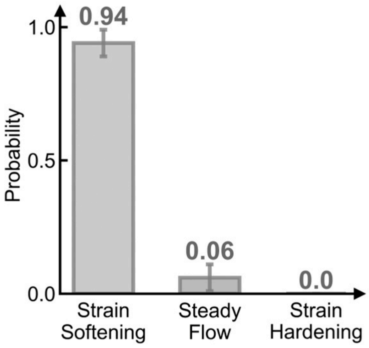

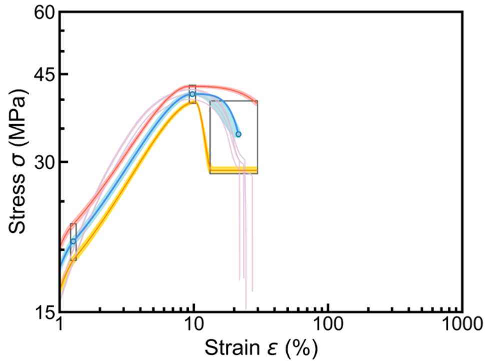

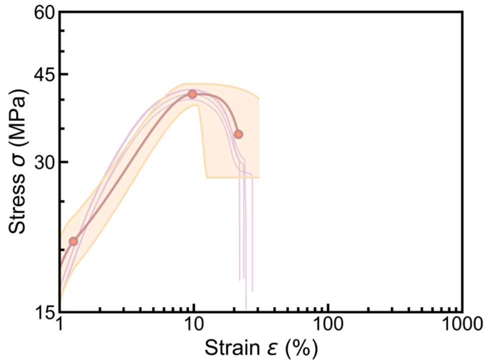

Sample 9:

[80, 80, 50, 24.858]

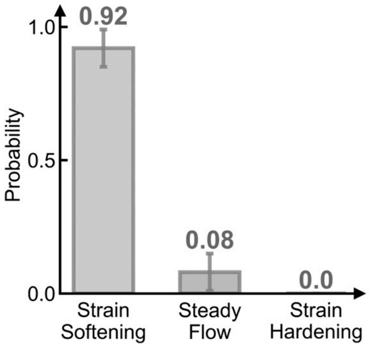

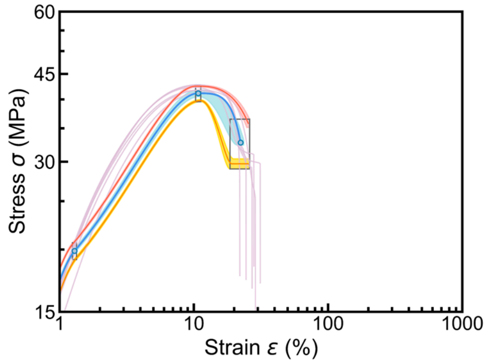

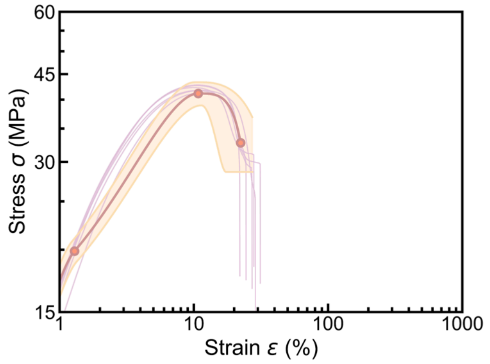

Sample 10:

[80, 20, 30, 41.43]

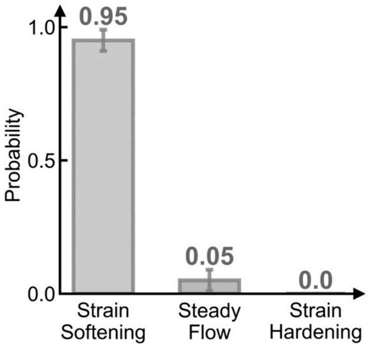

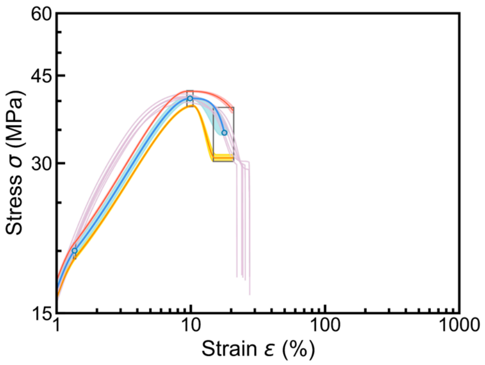

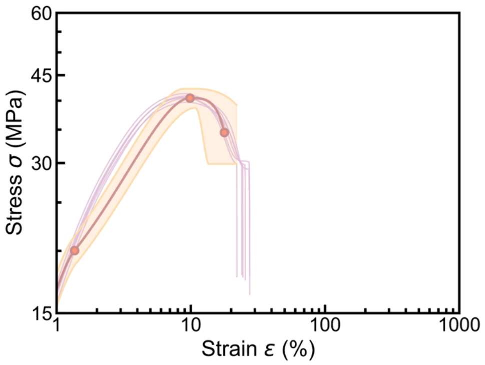

Sample 11:

[80, 50, 50, 41.43]

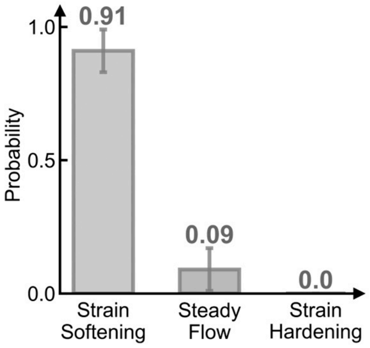

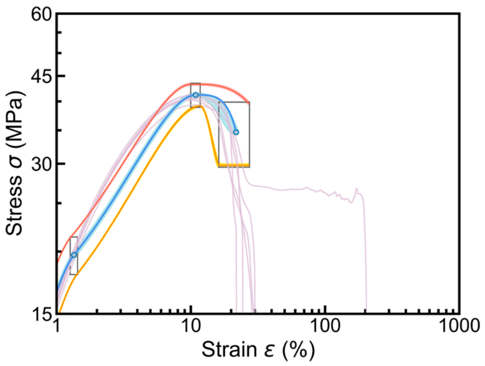

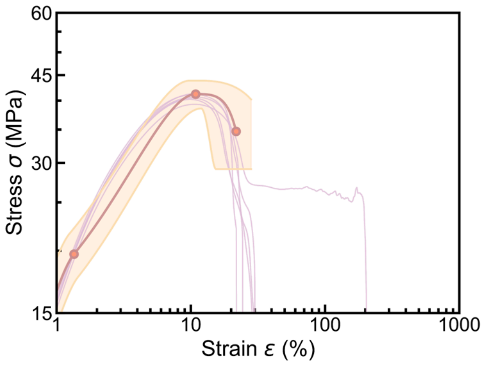

Sample 12:

[80, 80, 70, 41.43]

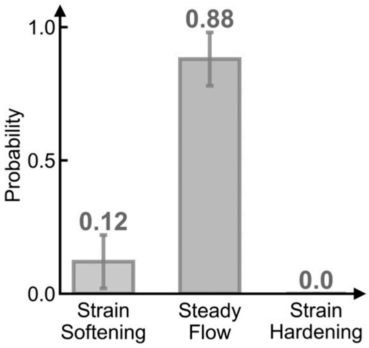

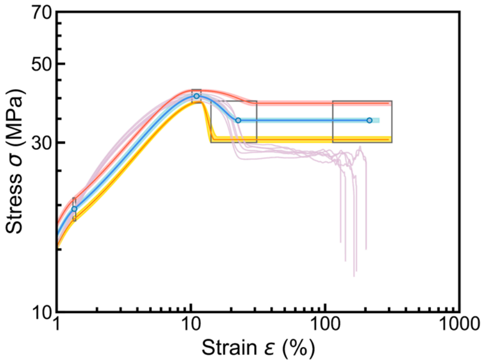

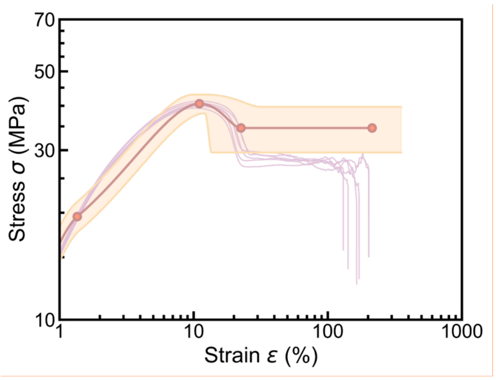

Sample 13:

[20, 20, 50, 41.43]

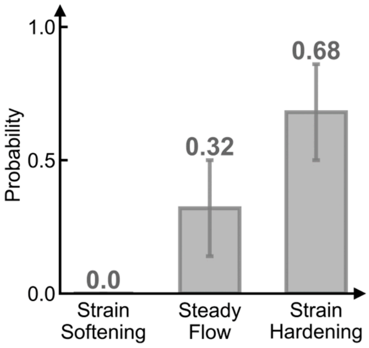

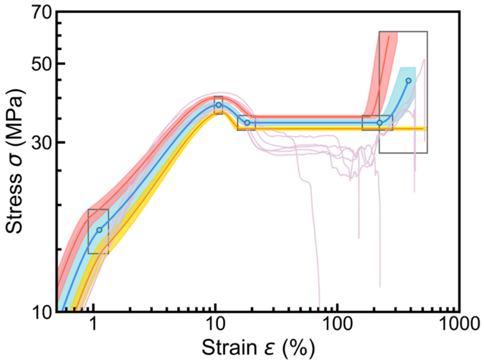

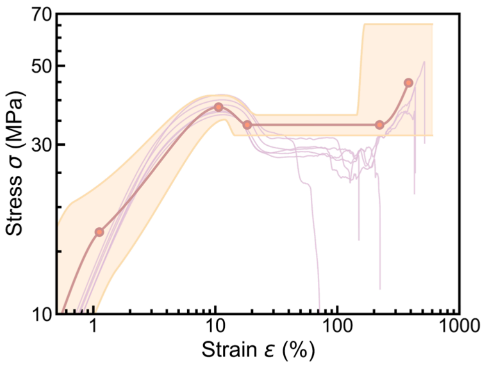

Sample 14:

[20, 50, 70, 41.43]

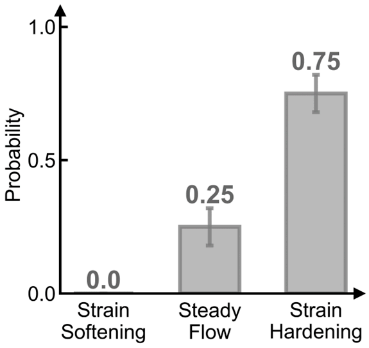

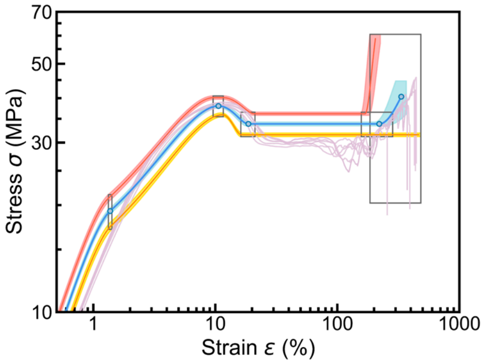

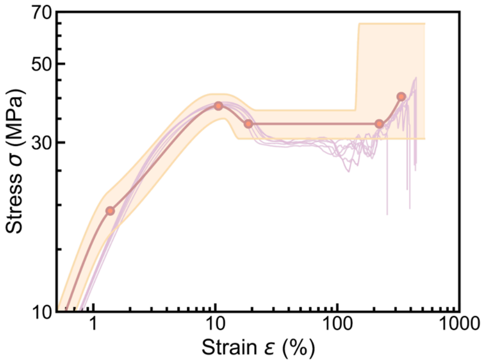

Sample 15:

[20, 80, 30, 41.43]

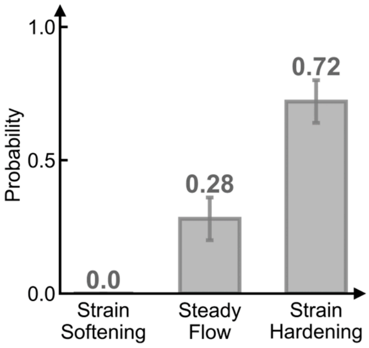

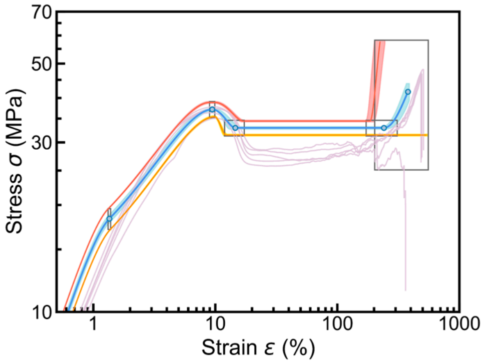

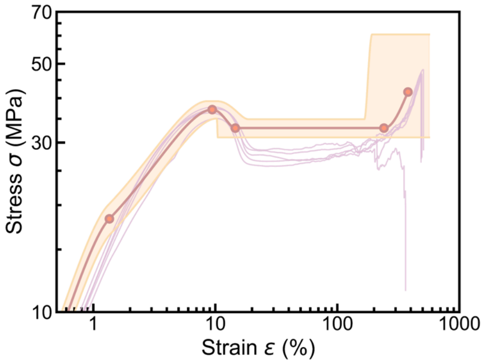

Sample 16:

[50, 20, 70, 41.43]

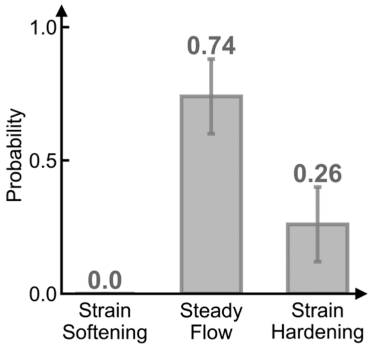

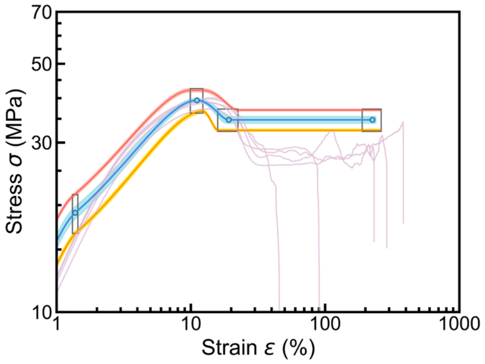

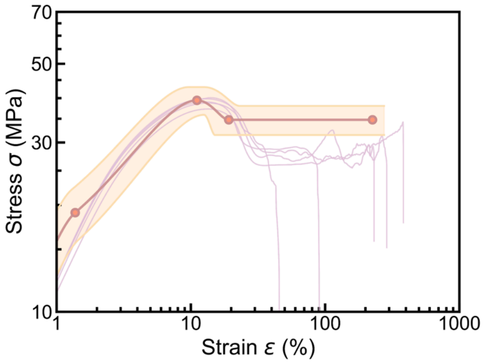

Sample 17:

[50, 50, 30, 41.43]

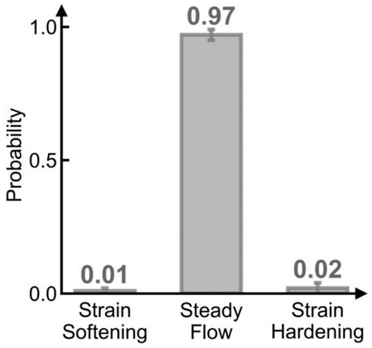

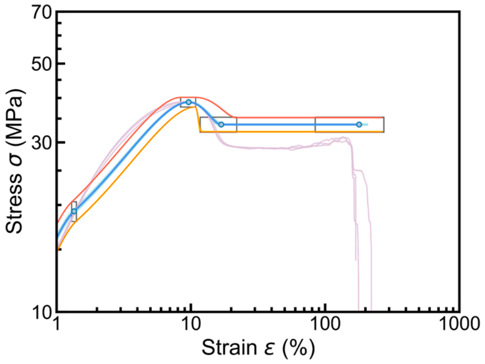

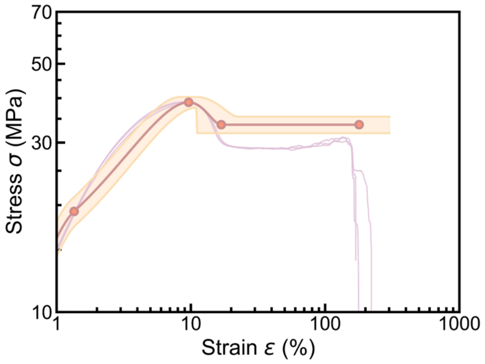

Sample 18: [50, 80, 50, 41.43]	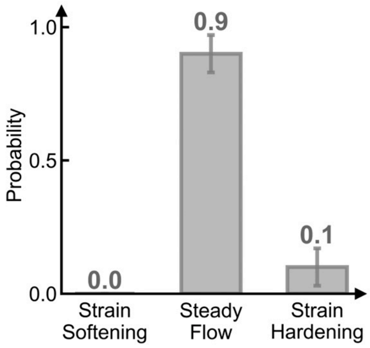	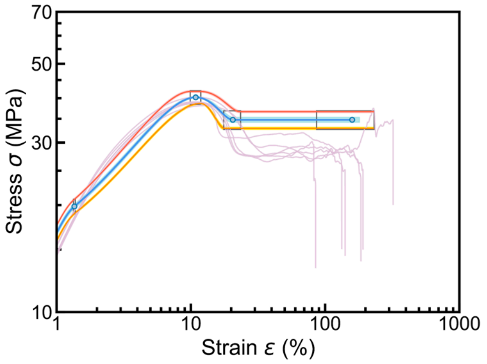 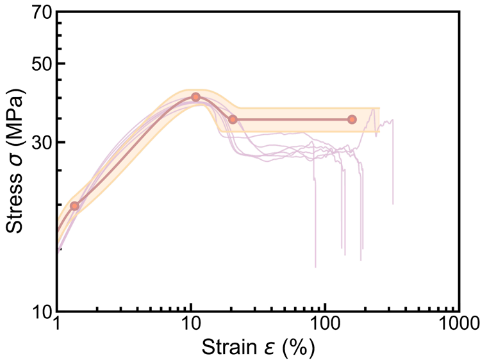
Sample 19: [50, 20, 30, 58.002]	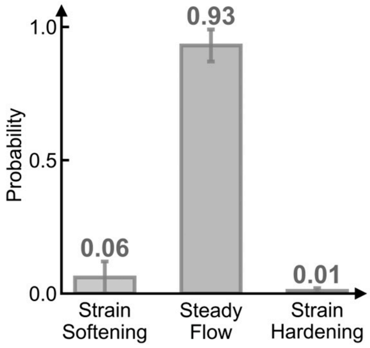	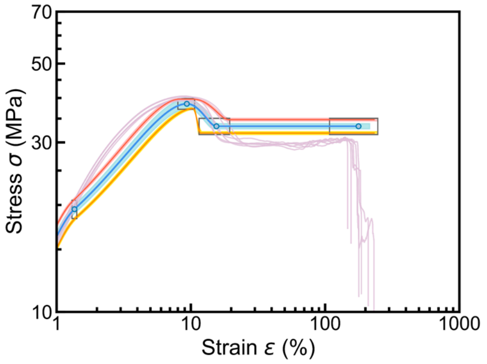 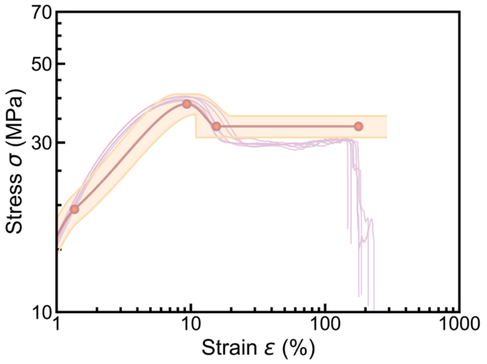

Sample 20:

[50, 50, 50, 58.002]

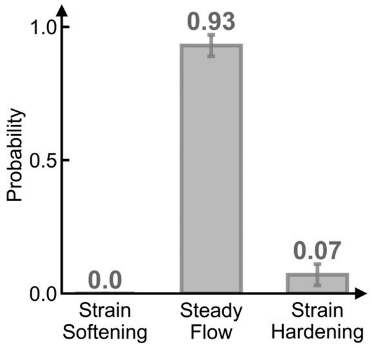

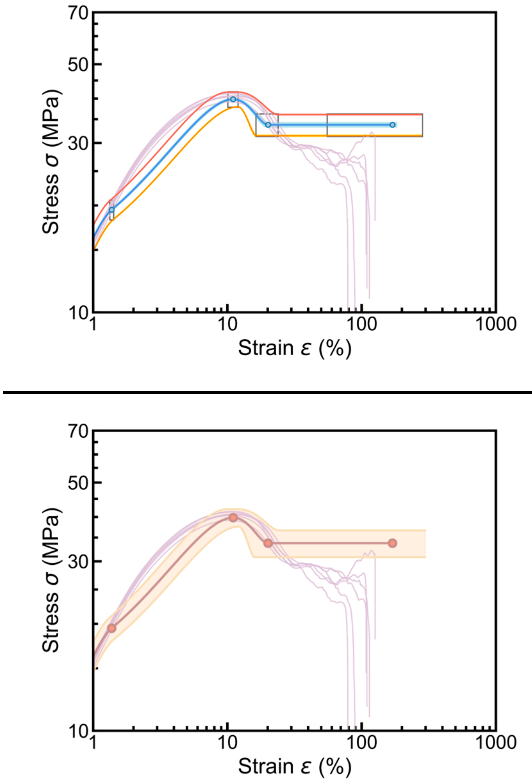

Sample 21:

[50, 80, 70, 58.002]

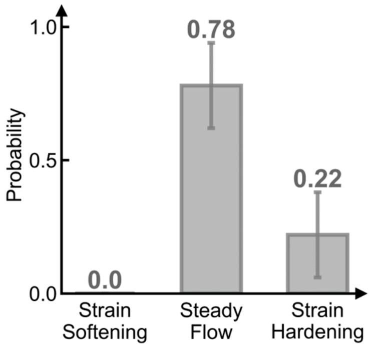

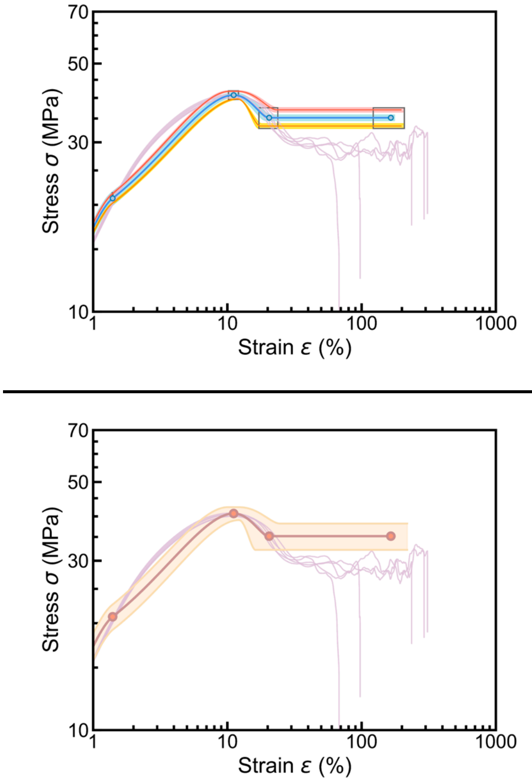

Sample 22:

[80, 20, 50, 58.002]

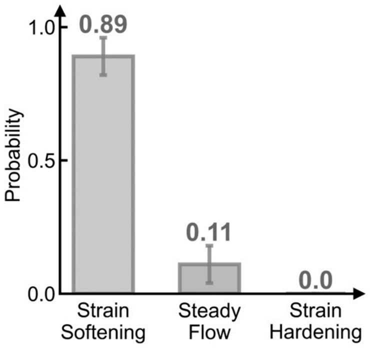

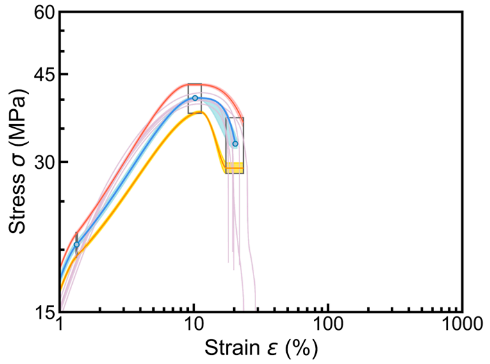

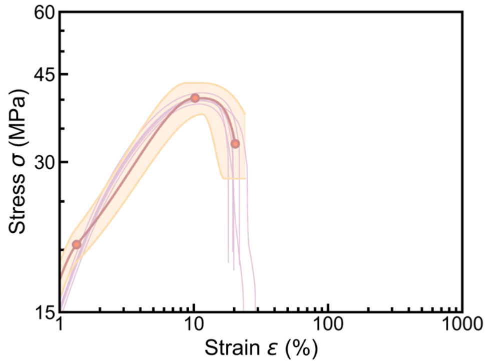

Sample 23:

[80, 50, 70, 58.002]

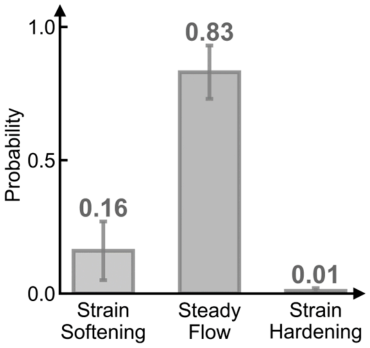

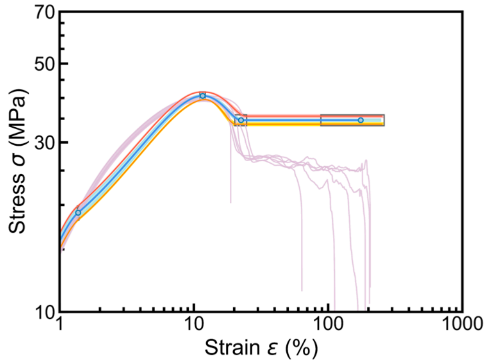

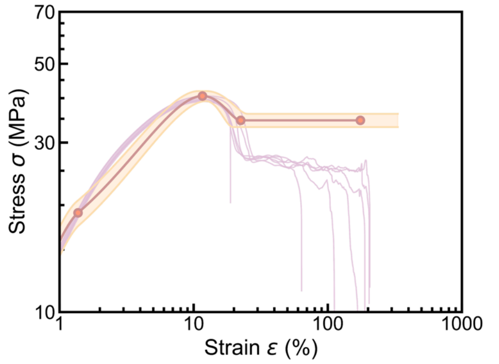

Sample 24:

[80, 80, 30, 58.002]

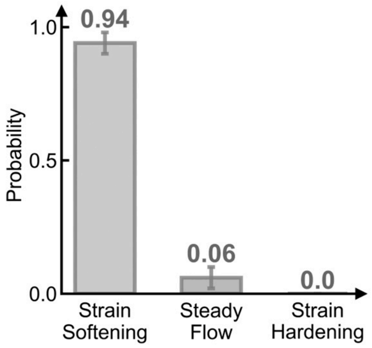

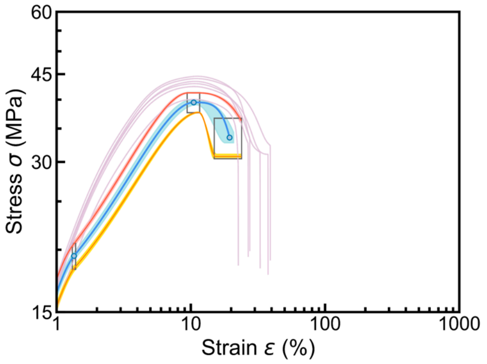

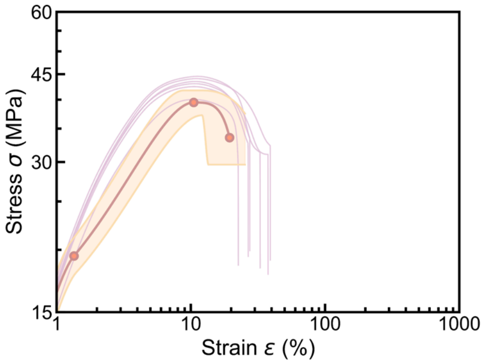

Sample 25:

[20, 20, 70, 58.002]

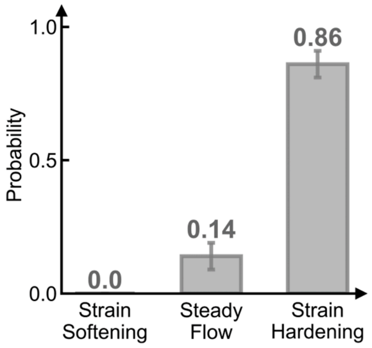

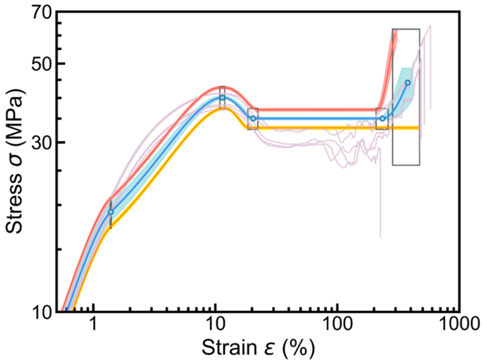

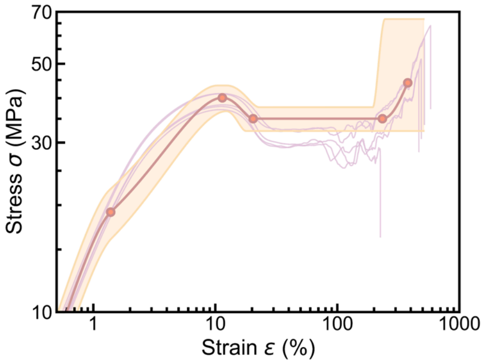

Sample 26:

[20, 50, 30, 58.002]

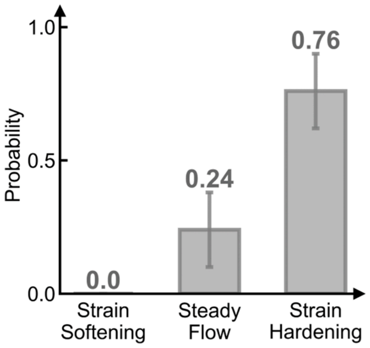

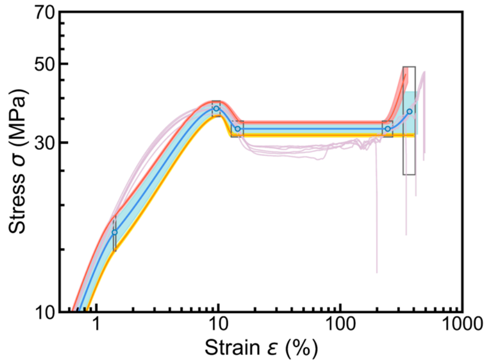

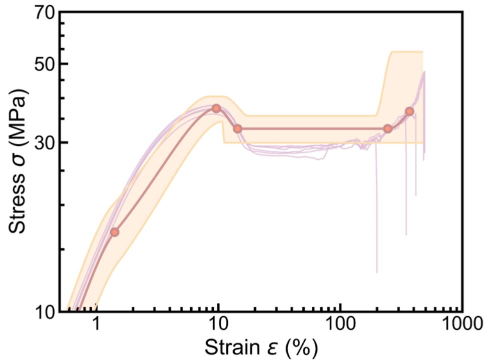

Sample 27:

[20, 80, 50, 58.002]

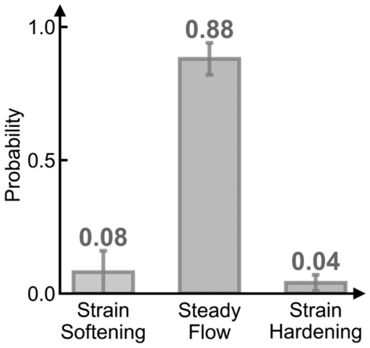

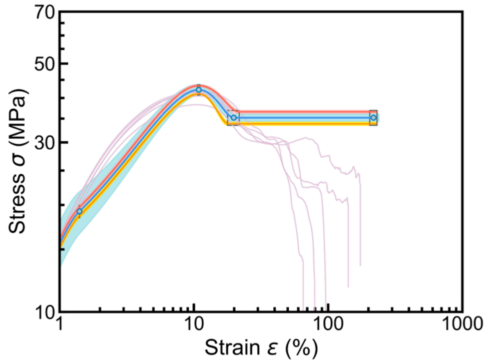

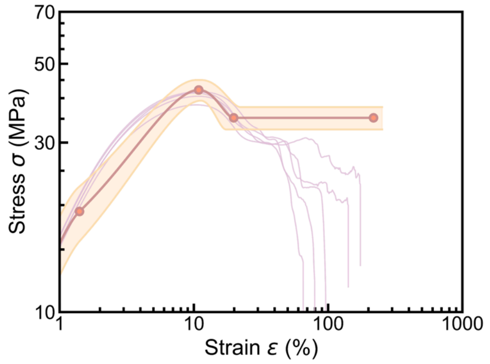

Table S2. List of 27 molding conditions used for prediction.

Test Samples	P_{inject} (MPa)	R_{inject} (cm^3/s)	P_{pack} (MPa)	T_{mold} (°C)
Sample 1	20	20	30	24.858
Sample 2	20	50	50	24.858
Sample 3	20	80	70	24.858
Sample 4	50	20	50	24.858
Sample 5	50	50	70	24.858
Sample 6	50	80	30	24.858
Sample 7	80	20	70	24.858
Sample 8	80	50	30	24.858
Sample 9	80	80	50	24.858
Sample 10	80	20	30	41.43
Sample 11	80	50	50	41.43
Sample 12	80	80	70	41.43
Sample 13	20	20	50	41.43
Sample 14	20	50	70	41.43
Sample 15	20	80	30	41.43
Sample 16	50	20	70	41.43
Sample 17	50	50	30	41.43
Sample 18	50	80	50	41.43
Sample 19	50	20	30	58.002
Sample 20	50	50	50	58.002
Sample 21	50	80	70	58.002
Sample 22	80	20	50	58.002
Sample 23	80	50	70	58.002
Sample 24	80	80	30	58.002
Sample 25	20	20	70	58.002
Sample 26	20	50	30	58.002
Sample 27	20	80	50	58.002

Reference

1. Liu, H.; Wu, F.-Y.; Zhong, G.-J.; Li, Z.-M. Predicting the complex stress-strain curves of polymeric solids by classification-embedded dual neural network. *Mater. Des.* 2023, 227, 111773. https://doi.org/10.1016/j.matdes.2023.111773.
2. Kamalov, F.; Moussa, S.; Reyes, J.A. Data Transformation in Machine Learning: Empirical Analysis. In Proceedings of the 2023 International Conference on Innovation and Intelligence for Informatics, Computing, and Technologies (3ICT), Sakheer, Bahrain, 20–21 November 2023; pp. 115–120. https://doi.org/10.1109/3ICT60104.2023.10391512.
3. Djordjević, L.; Jordović-Pavlović, M.I.; Ćojbašić, Ž.M.; Galović, S.P.; Popović, M.N.; Nešić, M.V.; Markushev, D.D. Influence of data scaling and normalization on overall neural network performances in photoacoustics. *Opt. Quant. Electron.* **2022**, *54*, 501. https://doi.org/10.1007/s11082-022-03799-1.
4. Mehta, P.; Bukov, M.; Wang, C.-H.; Day, A.G.R.; Richardson, C.; Fisher, C.K.; Schwab, D.J. A high-bias, low-variance introduction to Machine Learning for physicists. *Phys. Rep.* **2019**, *810*, 1–124. https://doi.org/10.1016/j.physrep.2019.03.001.
5. Hirschfeld, L.; Swanson, K.; Yang, K.; Barzilay, R.; Coley, C.W. Uncertainty Quantification Using Neural Networks for Molecular Property Prediction. *J. Chem. Inf. Model.* **2020**, *60*, 3770–3780. https://doi.org/10.1021/acs.jcim.0c00502.

Disclaimer/Publisher's Note: The statements, opinions and data contained in all publications are solely those of the individual author(s) and contributor(s) and not of MDPI and/or the editor(s). MDPI and/or the editor(s) disclaim responsibility for any injury to people or property resulting from any ideas, methods, instructions or products referred to in the content.
